# Resisting spread of environmental-pollution diseases due to Portland-cement industries: green nanoclay applications

**DOI:** 10.1038/s41598-023-27759-1

**Published:** 2023-01-09

**Authors:** Refat El-Sheikhy

**Affiliations:** Bughshan Research Chair in Expansive Clay Soil, Civil Engineering Department, College of Engineering, King Saud University, P.O. Box 800, Riyadh, 11421 Saudi Arabia

**Keywords:** Environmental sciences, Diseases, Engineering, Materials science, Nanoscience and technology

## Abstract

New eco-friendly thermal-casting ductile CPNC-concrete and CPNC-mortar materials have been developed without Portland-cement or water-use. It needs three hours for thermal synthesis. It contains 5%, 10% or 15% green natural nanoclay–polymer nanocomposite CPNC as a bonding-agent instead of Portland-cement for mixing with sand and crushed-stones by heating at 250 °C in electric ovens. CPNC is a dry powder consisting of 5% montmorillonite (MMT) nanoclay and 95% high-density polyethylene (HDPE). The study includes mixing, thermal synthesis, mechanical, fracture, and ductility property testing, and characterizations of the chemical composition, microstructure morphology and homogeneity using EDAX, SEM and XRD. It has new properties, such as a homogenous-microstructure with bonding, self-compaction abilities, no-cracks, ductility, good compressive and tensile strengths, and good fracture properties. It does not exhibit steel corrosion; it has rapid processing, no-water curing, low-density, no-shrinkage cracks, no-sudden failure, no-pollution utilizing a new manufacturing technique. CPNC-concrete is developed to stop the Portland-cement industry problems, to produce a clean and green environment without Portland-cement damage for human beings and to conserve drinking water. It has potential for different applications in construction, and it is suitable for 3D techniques with fast construction. Furthermore, this material requires new special codes for design and construction.

## Introduction

The dangerous environmental problems of Portland-cement industries affect human health^1–11^, deteriorate infrastructure^12–17^. Progresses in fields of nanoclay-based polymer nanocomposite science^18–30^, geotechnical engineering^31^, structural engineering and concrete structure construction^12–17^ suffer from serious problems due to using Portland-cement; these issues have encouraged us to try to solve the problems with concrete and Portland-cement by using natural green applications of nanocomposites instead of Portland-cement^1–11^. Therefore, this research idea is studied, and these results can solve Portland-cement problems and find new materials of suitable processing techniques and properties that lead to new construction techniques which will need new design codes to replace the existing design codes^12–17^ regarding Portland-cement materials and concrete construction fields. In this study, we develop a new application of nanoclay-based polymer nanocomposites^18–30,42–44^ as green nanocomposite cements to replace Portland-cement for concrete and construction industries. As a result of the study, changes between conventional Portland-cement concrete and recent green CPNC-concrete conclude main differences; the main important results are avoiding a large ratio of environmental pollution during the production and application of Portland-cement^31–34,36–40^, avoiding diseases caused by cement dust and preventing use of drinking water for the concrete construction industry, especially for countries with few water resources. Moreover, these results can minimize construction time and prevent steel corrosion due to the absence of water use. This solution will use green natural materials that do not cause pollution, it will use polymers that are inexpensive, and it may use recycled polymers to prevent millions of tons of polymers from entering the environment every year. Developed CPNC-concrete and construction materials have many other advantages in comparison to Portland-cement-based materials^31–34,36–40^, like ductility, avoiding sudden brittle failure and crushing, saving water-consumption, eliminating use of Portland-cement, eco-friendliness, and new applications.

## Results

Results of testing and characterization for investigating mechanical and fracture properties, chemical composition, microstructure morphology, bond and homogeneity of CPNC-concrete and CPNC-mortar are illustrated in figures and tables followed by detailed discussion.

### Materials

**Figure 1:**
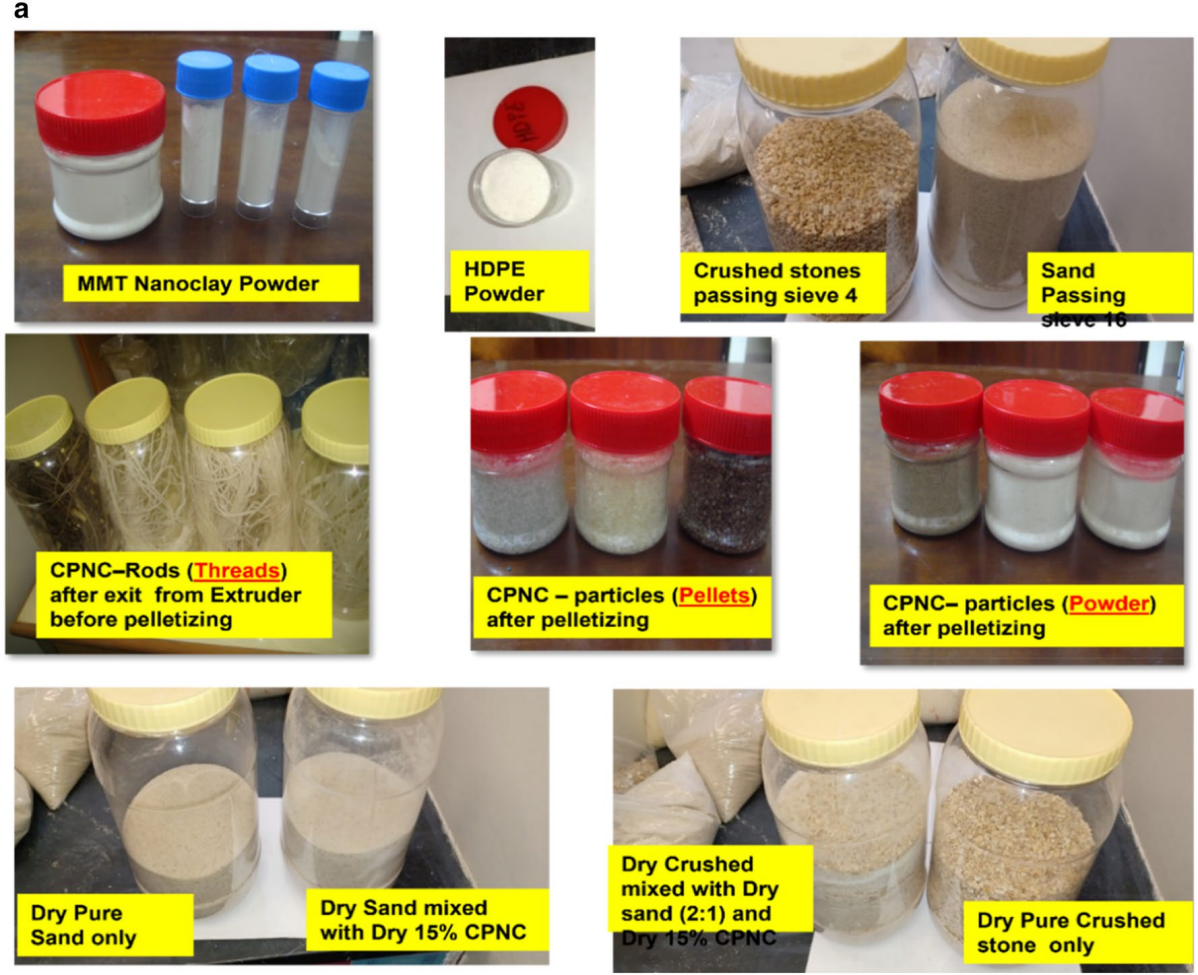

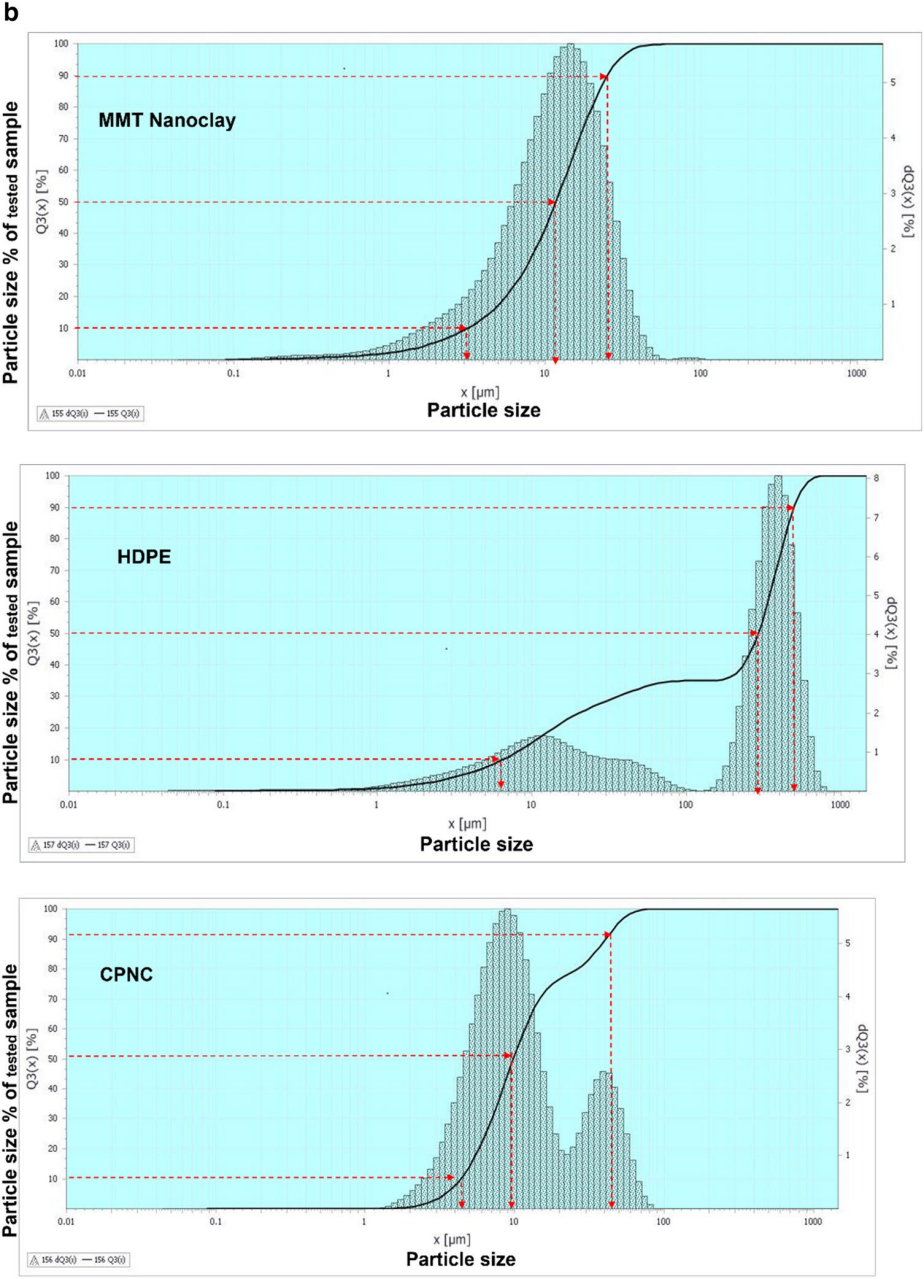

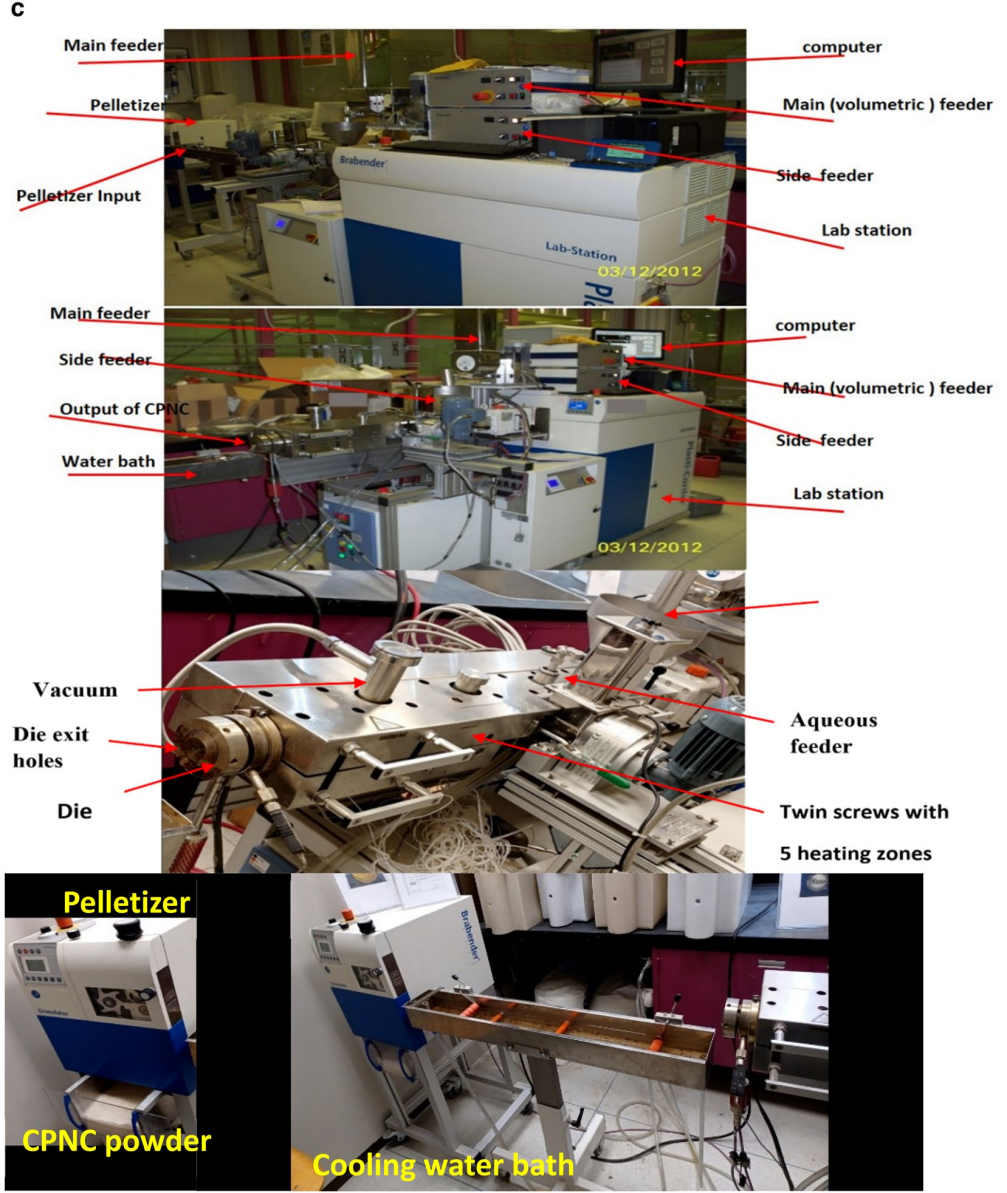

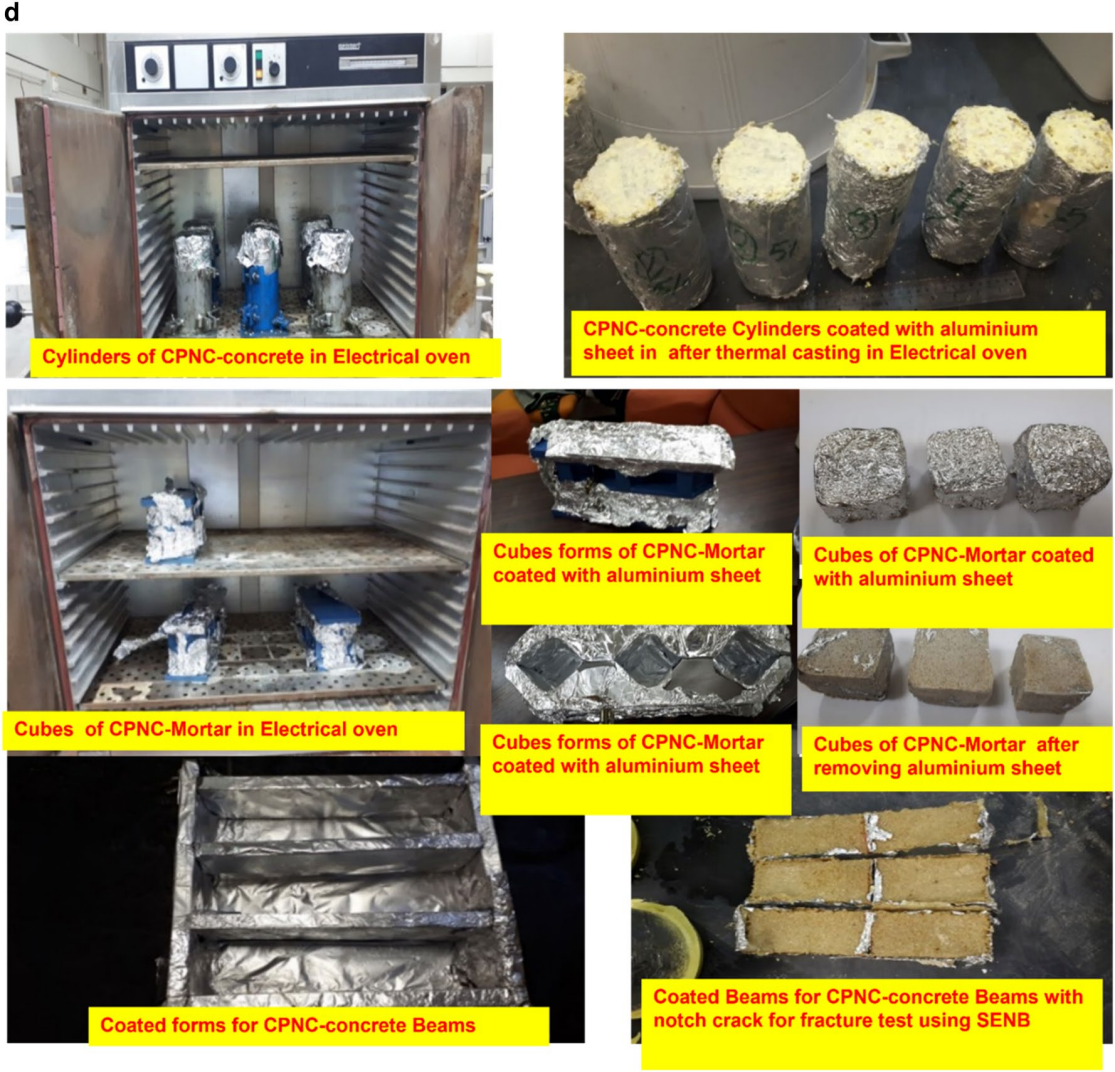

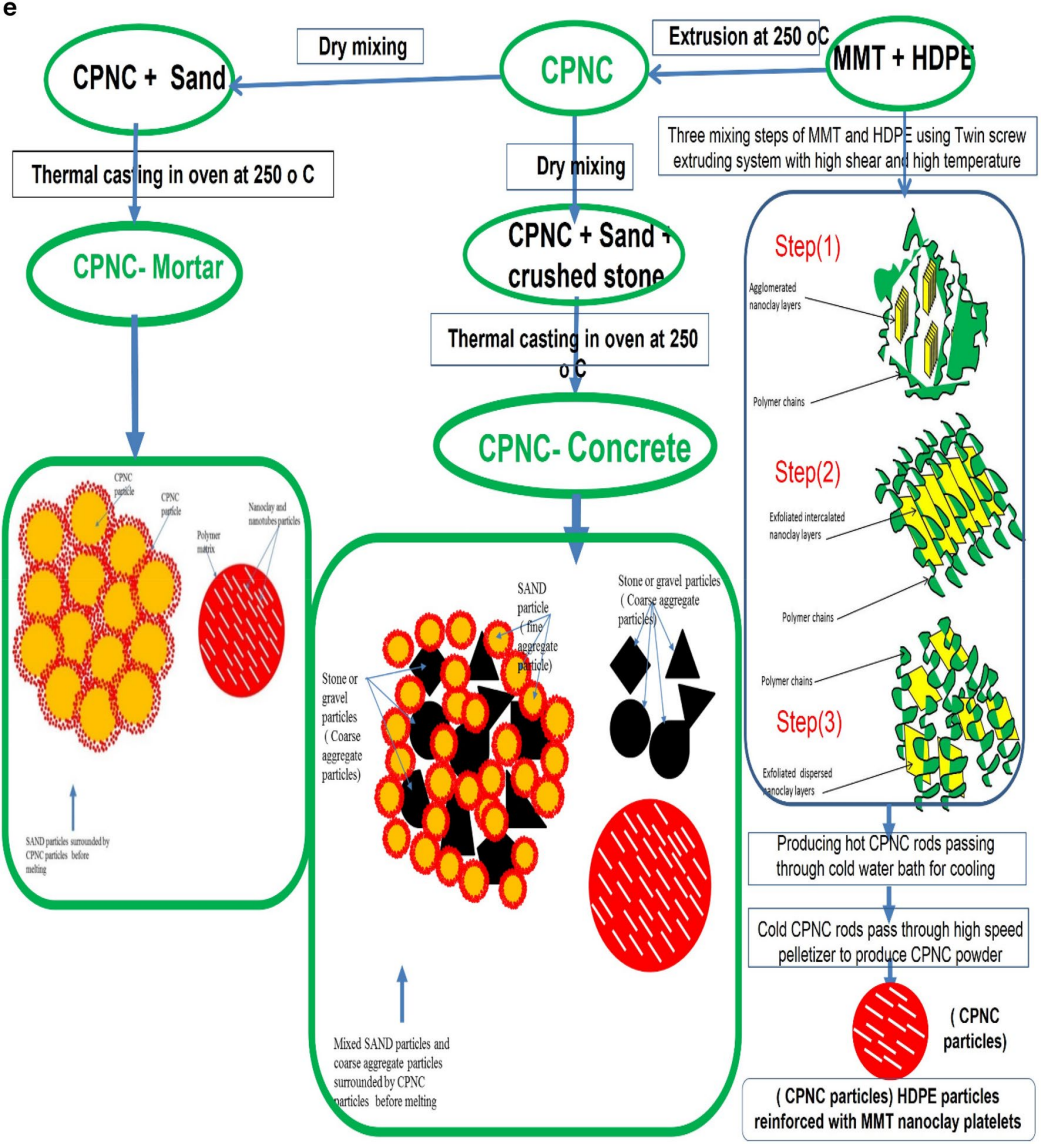

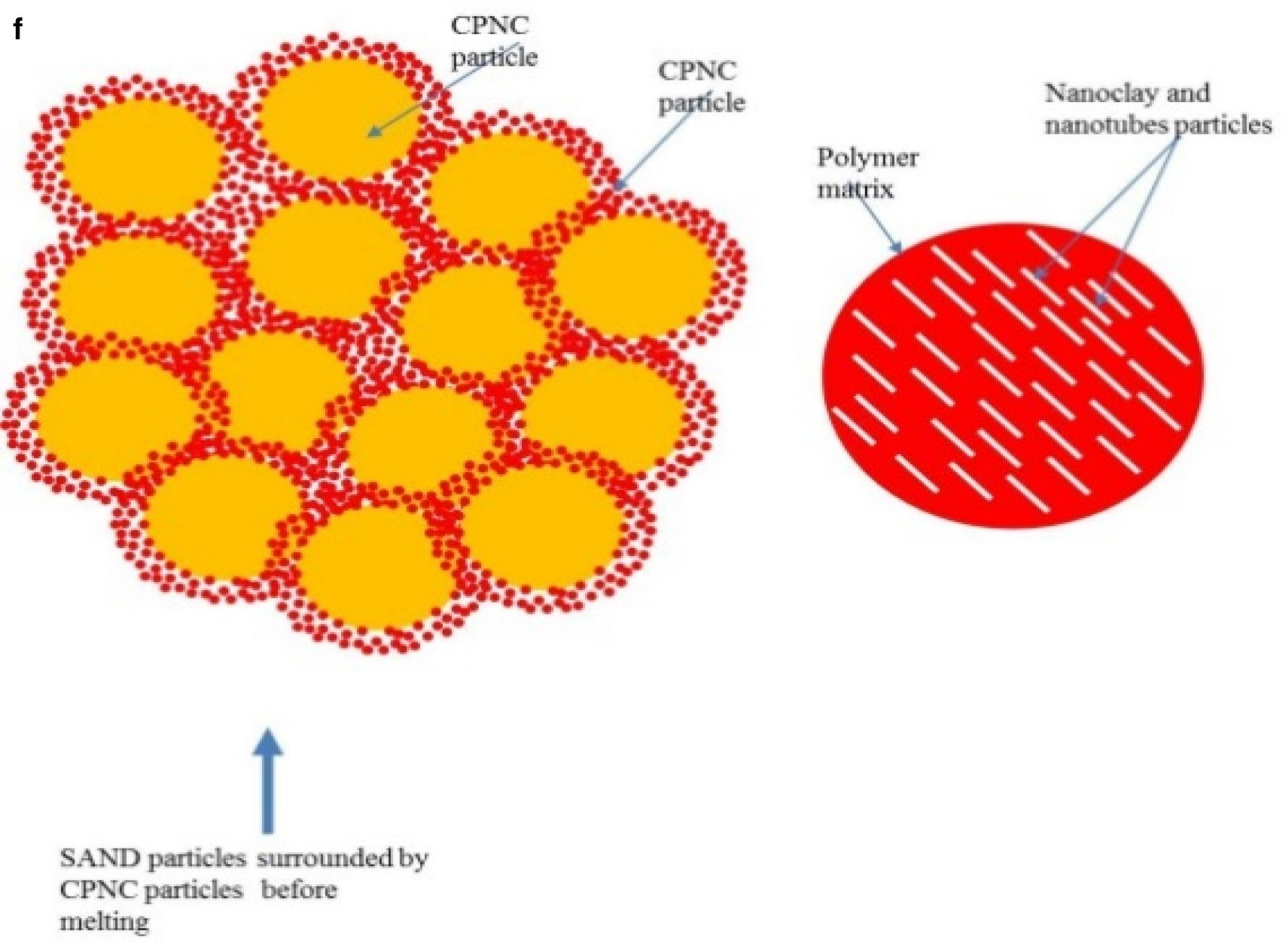
**(a)** Raw materials of MMT, HDPE, CPNC, CPNC-mortar and CPNC-concrete. CPNC is made of MMT and HDPE as hot rods; after cooling in a water bath, CPNC is converted into pellets and powder by a pelletizer before mixing with sand and crushed stones to produce CPNC concrete and CPNC-mortar. (**b)** Sieve analysis of raw materials of MMT nanoclay, HDPE, and CPNC by using a laser particle analyser indicating the particle size and percentage ratio in the quantity of each material; examples are shown at ratios of 10%, 50%, and 90%, where the HDPE particle size is larger than the agglomerated MMT particle size before melting in the extruder. After mixing under high temperature and high shear effect, MMT is exfoliated and dispersed in HDPE chains producing CPNC rods. Then, the produced powder of CPNC has a particle size less than that of the original HDPE and agglomerated MMT particles. (**c)** Extruding system for synthesizing CPNC from MMT and HDPE; the figure shows the controller lab station components, twin screw of 5 heating zones, die, feeders, cooling water bath and high-speed pelletizer, including the collector tank of the final CPNC powder. (**d)** Electric oven for thermal casting of CPNC concrete and CPNC mortar. Electric oven and moulds of cylinders covered with aluminium foil during thermal casting; some CPNC-concrete cylinders are coated with thin aluminium sheets after thermal casting. Electric oven and moulds of CPNC mortar cubes covered with aluminium foil during thermal casting, CPNC mortar cubes coated with thin aluminium sheets after thermal casting, and CPNC mortar cubes after removing the aluminium sheets. Moulds of CPNC-concrete notched beams coated with thin aluminium sheets, and samples of CPNC concrete notched beams for fracture properties test. **(e)** Flowchart of CPNC, CPNC concrete and CPNC mortar, showing the manufacturing steps of CPNC through synthesis by the thermal extruding of melted 95% HDPE powder with 5% agglomerated MMT; there is extruding under high-shear twin screws of five different thermal zones, including the feeding zone and die at 250 °C, to exfoliate MMT into nanoplatelets and disperse them between melted HDPE chains, making bonds and finally exiting at the die zone. Then, the material is converted to powder in the pelletizer. Then, CPNC powder is mixed with sand or sand and crushed stones to produce CPNC concrete and CPNC–mortar under thermal casting in electrical or microwave ovens. (**f)** Model of CPNC particles showing nanoclay sheet arrangement inside HDPE, CPNC-mortar and CPNC-concrete showing CPNC particle distribution around aggregate particles; the sizes are analysed by the wet method.

Results are shown in Table 1, and figures, where Fig. 1a, shows raw materials of MMT, HDPE, CPNC, CPNC–mortar and CPNC–concrete, CPNC is made of MMT and HDPE as hot rods; after cooling in a water bath, CPNC is converted into pellets and powder by pelletizer before mixing with sand and crushed stones to produce CPNC-concrete and CPNC-mortar, Fig. 1b, shows sieve analysis of raw materials of MMT nanoclay, HDPE, and CPNC by using a laser particle analyser indicating particle size and percentage ratio in quantity of each material; examples are shown at ratios of 10%, 50%, and 90%, where HDPE particle size is larger than agglomerated MMT particle size before melting in extruder. After mixing under high temperature and high shear effect, MMT is exfoliated and dispersed in HDPE chains producing CPNC rods. Then, produced powder of CPNC has a particle size less than that of original HDPE and agglomerated MMT particles. Figure 1c, shows extruding system for synthesizing CPNC from MMT and HDPE; it shows lab-station components, twin-screw of five heating zones, die, feeders, cooling water-bath and high-speed pelletizer with collector of CPNC powder. Figure 1d, shows electric oven for thermal casting of CPNC-concrete and CPNC-mortar, electric oven and moulds of cylinders covered with aluminium foil during thermal casting; some CPNC–concrete cylinders are coated with thin aluminium sheets after thermal casting, electric oven and moulds of CPNC-mortar cubes covered with aluminium foil during thermal casting, CPNC-mortar cubes coated with thin aluminium sheets after thermal casting, and CPNC-mortar cubes after removing aluminium sheets in addition to moulds of CPNC-concrete notched beams coated with thin aluminium sheets, and samples of CPNC-concrete notched beams for fracture properties test. Figure 1e, is flowchart of CPNC, CPNC-concrete and CPNC-mortar, showing manufacturing steps of CPNC through synthesis by thermal extruding of melted 95% HDPE powder with 5% agglomerated MMT; there is extruding under high-shear twin screws of five different thermal zones, including feeding zone and die at 250 °C, to exfoliate MMT into nanoplatelets and disperse them between melted HDPE chains, making bonds and finally exiting at die zone. Then, it is converted to powder in pelletizer. CPNC powder is mixed with sand or sand and crushed-stones to produce CPNC-concrete and CPNC–mortar under thermal casting in electrical or microwave ovens. Figure 1f, is model of CPNC particles showing MMT particles arrangement inside HDPE, CPNC–mortar and CPNC–concrete showing CPNC particle distribution around aggregate particles; sizes are analysed by wet method.

**Table 1:** Properties of MMT (I.30T) nanoclay, HDPE (M40060), and CPNC made of both.

Material	Property		Value	Unit
Nanoclay (MMT I.30T)	Density		200	kg/m^3^
	Purity		99%	
	Dimensions	Thickness	1.0	nm
		Width	100	nm
		Length	400	nm
	Surface area		800	m^2^/g
HDPE (M40060)	Melting point		190	˚C
	Density at 23 ˚C		960	kg/m^3^
	Tensile strength at break		16	MPa
	Elongation		560	%
	Young’s modulus (E)		712	MPa
	Critical stress intensity factor (K_Ic_)		38	MPa (mm)^0.5^
	Fracture energy (G_f_ = K_I_^2^/E)		1.85	MPa (mm)
CPNC = (5%MMT + 95% HDPE)	Tensile strength at break		23	MPa
	Elongation		28	%
	Young’s modulus (E)		395	MPa
	Tensile strength at failure (fracture strength)		16.7	MPa
	Critical stress intensity factor (K_Ic_)		42	MPa (mm)^0.5^
	Fracture energy (G_f_ = K_I_^2^/E)		4.73	MPa (mm)

### Mechanical properties

**Figure 2:**
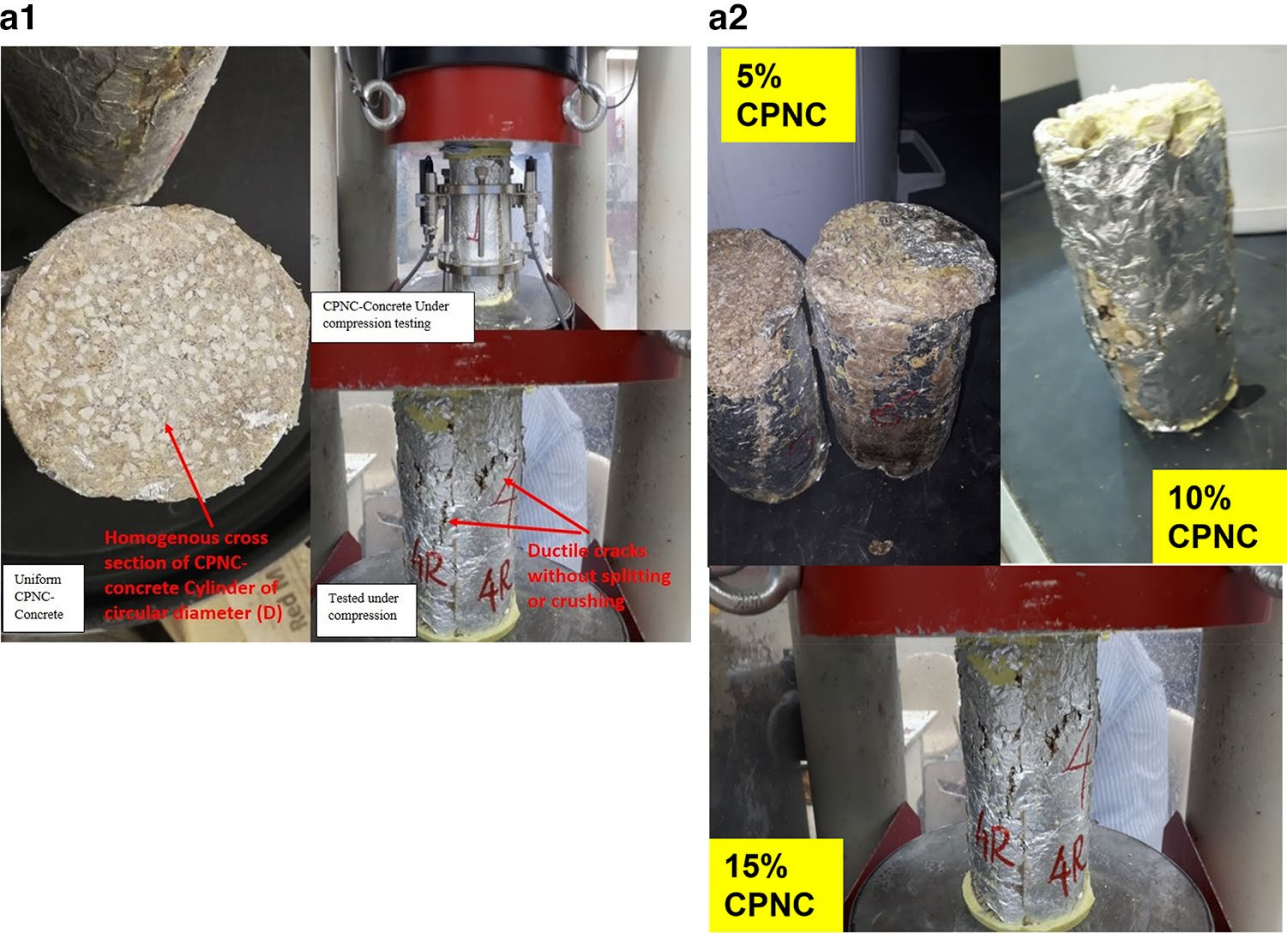

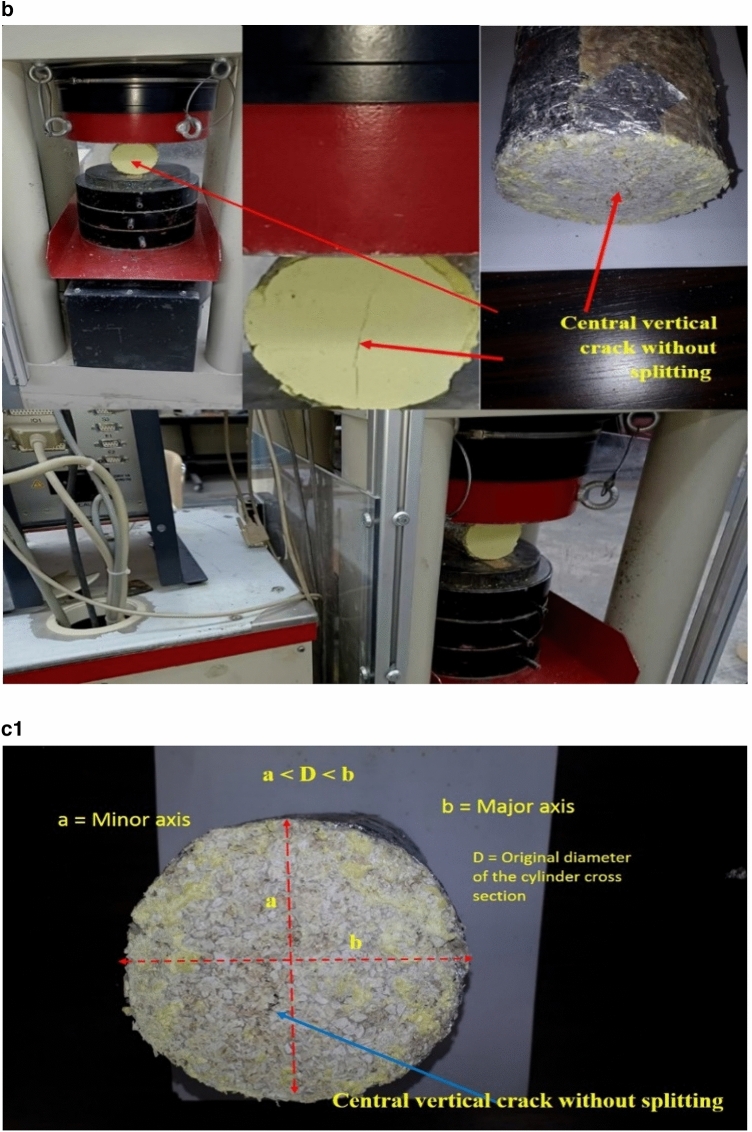

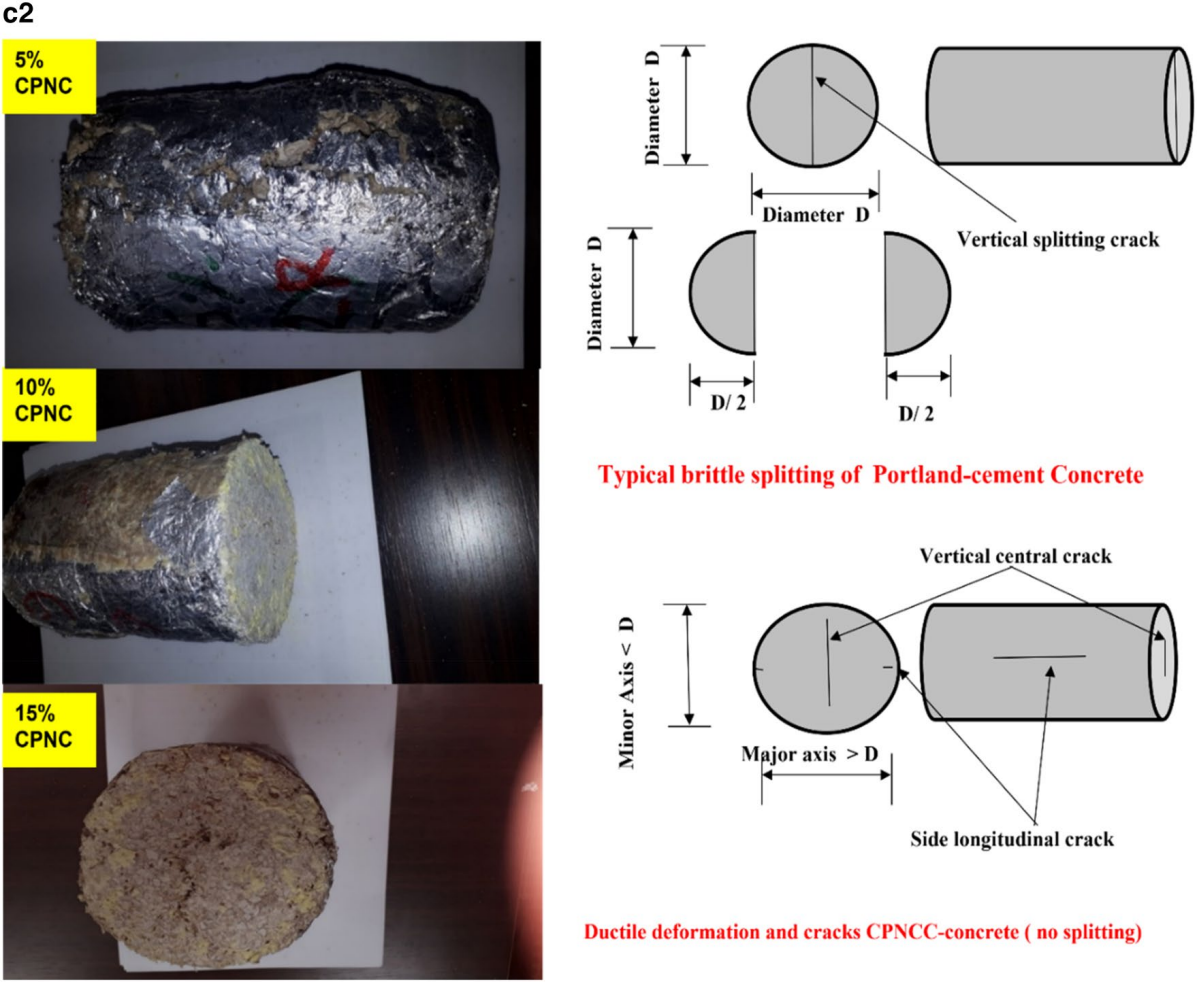
**(a1)** Compression test of CPNC-Concrete cylinders, Homogeneous cross-section without defects, and ductile failure without crushing. (**a2)** Testing of CPNC cylinders under unconfined compressive loading showing ductile failure modes of tested samples with 5%, 10%, and 15% CPNC ratios. There are no splitting or crushing failure modes, similar to Portland cement concrete. The cracking is ductile deformation without sudden failure. (**b)** Tensile test of CPNC concrete cylinders showing central cracking without splitting failure, proving ductility. (**c1)** Ductile deformation with vertical cracking without the splitting or brittle failure of tensile test cylinder sample of CPNC-concrete, showing changes in the vertical and horizontal diameters a and b, respectively, proving ductility. (**c2)** Cracking mode of CPNC concrete tensile strength test without splitting with different CPNC ratios (5%, 10%, and 15% CPNC) relative to Portland cement concrete splitting under compressive loading based on the ASTM standard, as shown in (**b,c1**), proving that CPNC concrete is ductile without sudden failure or crushing. Additionally, the typical failure mode of Portland cement concrete cylinders under compressive loading is shown in the figure for the tensile strength test; the cylinders fracture, and the failure is splitting at the vertical centreline of the cross section. In recent CPNC concrete, there is no splitting, sudden cracking or crack propagation; instead, there is ductile deformation of the cross section of the cylinder, as shown in the (**b,c1,c2**).

Mechanical properties testing includes both of compressive (Table 2) and tensile tests (Table 3) and Fig. 2, where Fig. 2a1, shows compression test of CPNC-Concrete cylinders, homogeneous cross-section without defects, and ductile failure without crushing, Fig. 2a2, shows testing of CPNC cylinders under unconfined compressive loading showing ductile failure modes of tested samples with 5%, 10%, and 15% CPNC ratios. There is no splitting or crushing failure modes, like Portland-cement concrete. Cracking is ductile deformation without sudden failure.

**Table 2:** Compressive strength properties, mechanical behaviours, and failure modes of CPNC concrete and CPNC mortar relative to those of Portland cement concrete; the results show big differences as CPNC concrete behaviour is lightweight and ductile without sudden failure or crushing, unlike Portland cement concrete.

Test type	Material	CPNC ratio%	Compaction ratio %	Density (γ) g/cm^3^	Modulus of elasticity (E) MPa	Compressive strength (σ) MPa	Mechanical behaviour	Standard deviation	Failure mode
Compressive strength test	CPNC concrete	5%	5	1.5	106	6	Ductile	0.11	Ductile deformation without splitting or crushing
		10%	7	1.6	150	7.5	Ductile	0.136	
		15%	19.6	1.75	285	23.4	Ductile	0.125	
	CPNC mortar	5%	0.0	1.24	106	5	Ductile	0.1	Ductile deformation without splitting or crushing
		10%	10	1.34	150	6.55	Ductile	0.15	
		15%	25	1.418	285	10.22	Ductile	0.145	
Comparison to Portland cement concrete	Portland cement concrete	Non	Non	2.5	> 14.0–50 GPa	> 25	Brittle		Fast brittle crack propagation with splitting and sudden crushing

**Table 3:** Test results of the tensile strength properties of CPNC concrete and CPNC mortar relative to Portland cement concrete proving that CPNC-materials are ductile and that the failure is not splitting or brittle failure; instead, it is ductile deformation without material separation or sudden propagation. The material is lightweight concrete.

Test type	Material	CPNC ratio %	Self-compaction ratio %	Density (γ) g/cm^3^	Tensile strength (MPa)	Standard deviation	Mechanical behaviour	Failure mode
Tensile strength test	CPNC concrete	5	5	1.5	2.54	0.15	Ductile	Ductile deformation without splitting or crushing
		10	7	1.6	2.61	0.14	Ductile	
		15	19.6	1.75	2.52	0.12	Ductile	
Comparison	Portland cement concrete	None	None	Heavy 2.5	2.5		Brittle	Fast brittle splitting and vertical crack propagation

Regarding tensile strength test, Fig. 2b, represents tensile test of CPNC-concrete cylinders showing central cracking without splitting failure, proving ductility while Fig. 2c1, shows ductile deformation with vertical cracking without splitting or brittle failure of tensile test cylinder sample of CPNC-concrete, showing changes in vertical and horizontal diameters a and b, respectively, and proving ductility. Fig. 2c2, is representing cracking mode of CPNC-concrete; under tensile strength test without splitting; with different CPNC ratios (5%, 10%, and 15% CPNC) relative to Portland-cement concrete splitting under compressive loading based on ASTM^14,15^, as shown in Fig. 2b,c1 proving that CPNC concrete is ductile without sudden failure or crushing. Additionally, typical known failure mode of Portland-cement concrete cylinders under compressive loading is shown for tensile strength test; cylinders failure is splitting at vertical centreline of cross-section while in CPNC-concrete, there is no-splitting cracking; instead, there is ductile deformation, as in Fig. 2b,c1,c2.

**Figure 3:**
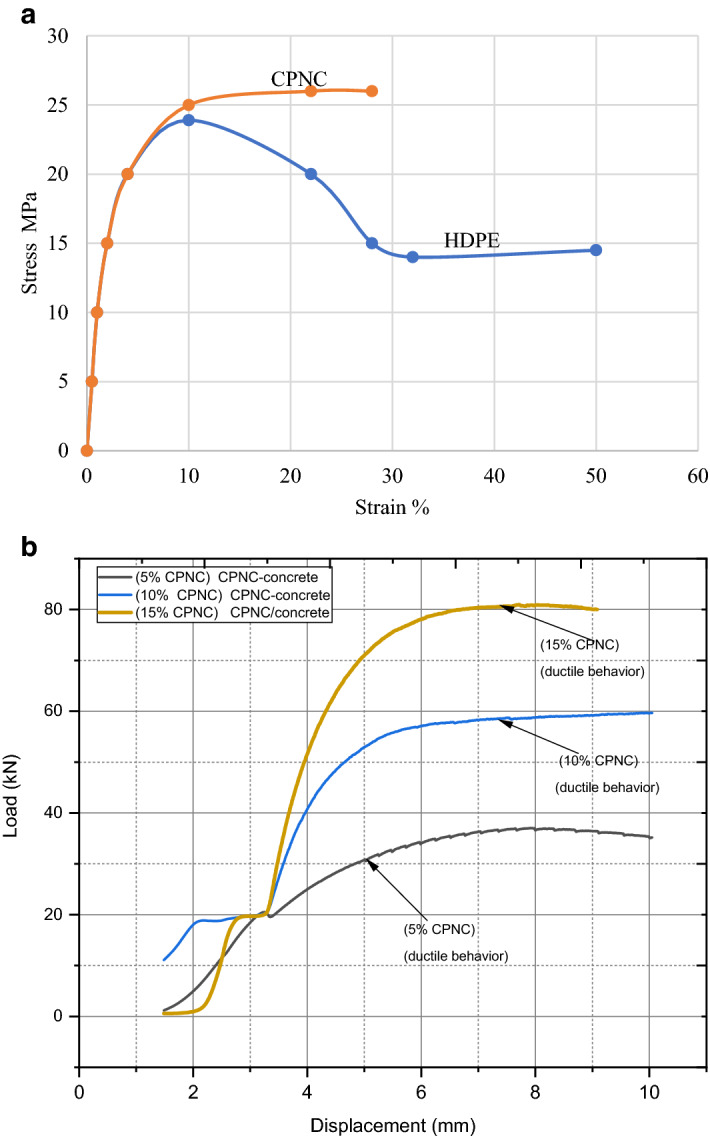
**(a)** Stress‒strain curves for the tensile test, showing changes between pure HDPE (M40060) and CPNC (HDPE and MMT) behaviours. (**b)** Load‒displacement curves of the compression test of CPNC concrete, showing ductile behaviour.

Regarding mechanical properties testing of HDPE and CPNC, Fig. 3a, represents stress‒strain relation for tensile test, showing changes between HDPE and CPNC behaviours, while Fig. 3b, shows load‒displacement ductile behaviour of compression test of CPNC-concrete.

### Fracture properties

**Figure 4:**
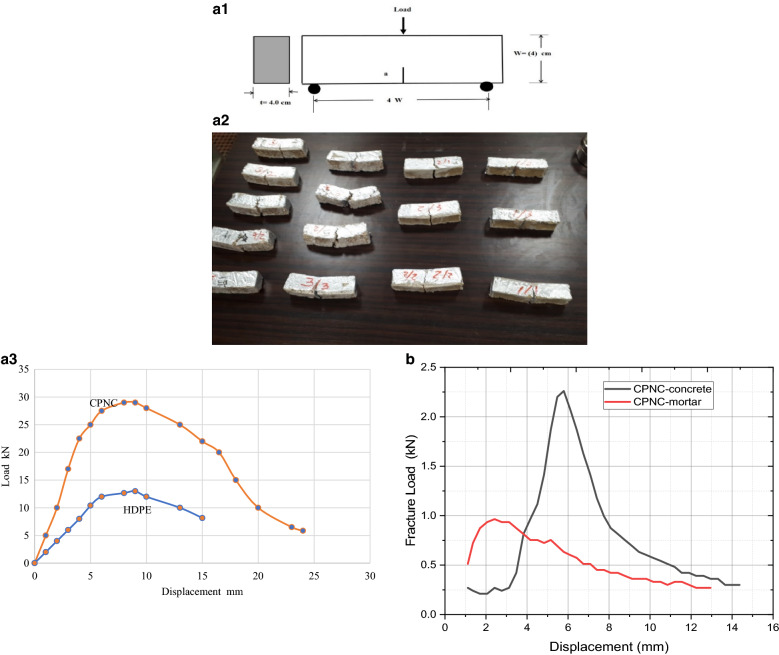
**(a1)** Standard model of SENB tested samples for fracture properties under 3-point loading, based on the ASTM standard. Some tested samples are shown in (**a2**). (**a2)** Tested samples of CPNC concrete for fracture properties, showing no separation at the cracked zone because there is no sudden failure or rapid crack propagation due to the ductile behaviour. (**a3)** Load‒displacement relationship of pure HDPE (M40060) and CPNC (HDPE and MMT) under the SENB fracture properties test. (**b**) Load‒displacement curves of tested beams of CPNC concrete and mortar for fracture toughness (K_Ic_) and fracture energy.

Fracture properties test results of HDPE and CPNC are shown in Table 1, while Table 4, is showing fracture properties of CPNC-concrete and CPNC-mortar. Figure 4a1, is showing standard model of SENB tested samples for fracture properties under 3-point loading, based on ASTM standard^12–17,41,45^. Some tested samples of CPNC-concrete are shown in Fig. 4a2, where no separation at cracked zone because there is no sudden failure or rapid crack propagation due to ductile behaviour. Figure 4a3, represents load‒displacement relationship for fracture properties test o under SENB, for pure HDPE (M40060) and CPNC, where Fig. 4b, shows load‒displacement curves of tested beams of CPNC-concrete and CPNC-mortar for testing of fracture toughness (K_Ic_) and fracture energy G_f_.

**Table 4:** Fracture properties of CPNC concrete and CPNC mortar relative to Portland cement materials.

Test type	Material	CPNC ratio %	Fracture toughness Κ_Ιc_ in kN (mm)^0.5^	Fracture energy (G_f_) (kN mm)	Standard deviation	Mechanical behaviour	Failure mode
Fracture property test	CPMC-mortar	5	2.184 kN (mm)^0.5^	689.0 (kN mm)	0.1	Ductile (Elastic‒plastic; no separation at crack vicinity)	Ductile deformation without splitting or crushing
		10	2.62 kN (mm)^0.5^	995.0 (kN mm)	0.1		
		15	2.916 kN (mm)^0.5^	1219.0 (kN mm)	0.1		
	CPNC concrete	5	3.46 kN (mm)^0.5^	1735.0 (kN mm)	0.1	Elastic–plastic (no separation at crack tip)	Ductile deformation without splitting or crushing
		10	4.0 kN (mm)^0.5^	2320 (kN mm)	0.1		
		15	8.2 kN (mm)^0.5^	9750.0 (kN mm)	0.1		
Comparison	Cement mortar	Non	0.5–1.5 MPa m^0.5^ (reference # 45)			Brittle (Elastic; usually, rapid propagation and separation at crack tip zone)	Fast brittle crack propagation, splitting and sudden crushing
	Cement concrete	Non	0.7 MN/m ^1.5^ (reference # 45)	16.0 Nm/m^2^ (reference # 45)			

The cracking mode found from the CPNC concrete tensile fracture test shows CPNC concrete and CPNC mortar with different CPNC ratios (5%, 10%, and 15% CPNC) relative to Portland cement concrete under 3-point loading for pre-cracked beams with notches; the results are shown in this table and Fig. 4a1–a3,b based on ASTM standards, and they prove that CPNC concrete is ductile without sudden failure or crushing.

### Characterizations

#### Chemical composition with EDAX

Chemical composition elements are shown in Table 5, for each of MMT, HDPE, CPNC, CPNC-mortar and CPNC-concrete.

**Table 5:** EDAX chemical compositions of CPNC, CPNC mortar, and CPNC concrete.

EDAX analysis	Element	CPNC (5% MMT + 95% HDPE)	CPNC mortar (15% CPNC)	(Sand + coarse aggregate) + CPNC (15% CPNC)
Elements by wt.%	C	54.0		
	O	28.4	54.7	51.5
	Si	7.8	17.8	27.6
	Al	1.4	7.6	8.7
	Mg		1.9	3.7
	Fe	0.4	4.2	3.2
	K		2.0	2.3
	Cu		0.9	1.1
	Zn	7.7	0.7	0.8
	Ca	0.2	9.4	0.8
	Ti		0.3	0.2
	Cl	0.1	0.5	0.1
Total 100%		100%	100%	100%

#### SEM

**Figure 5:**
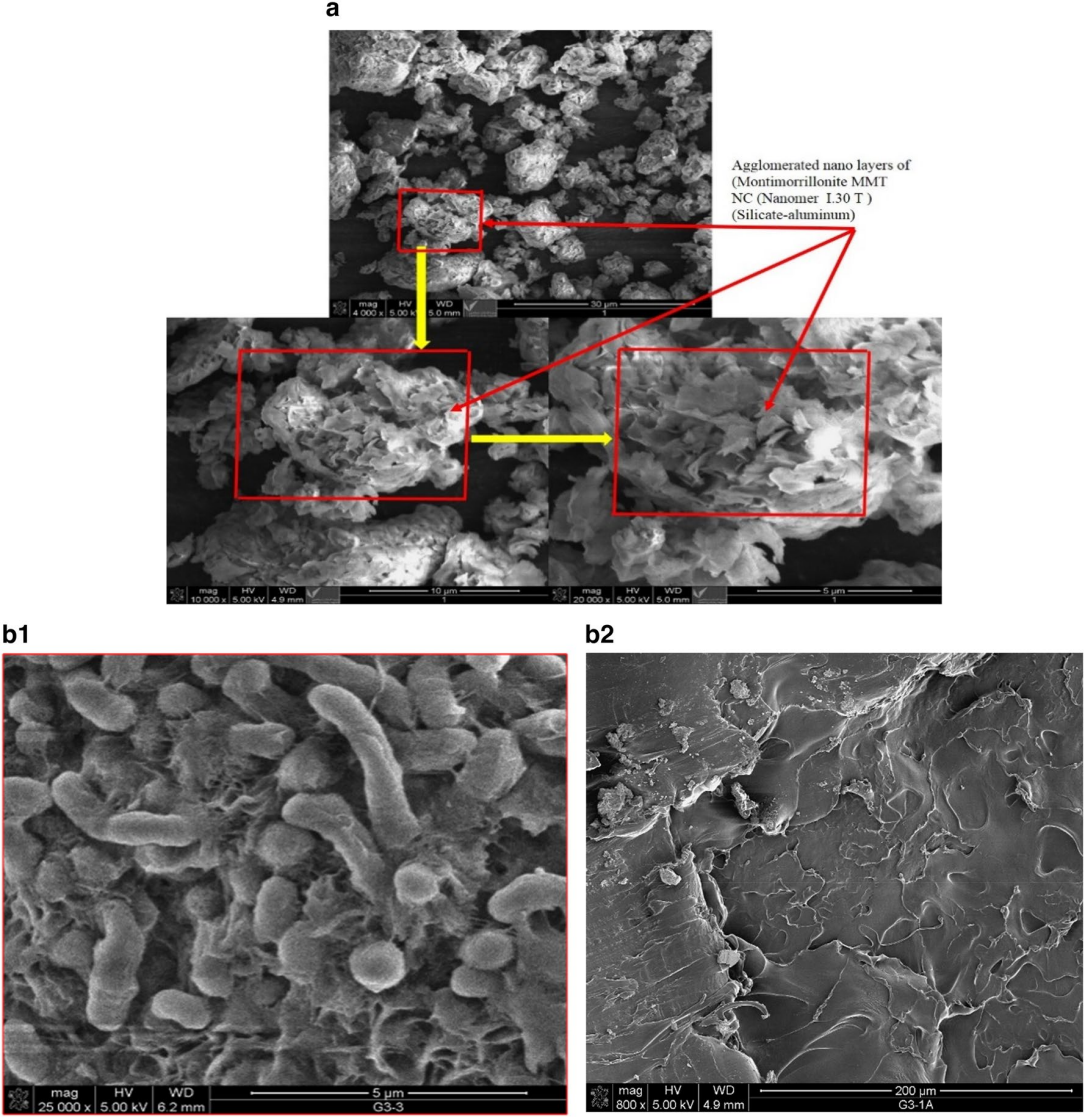

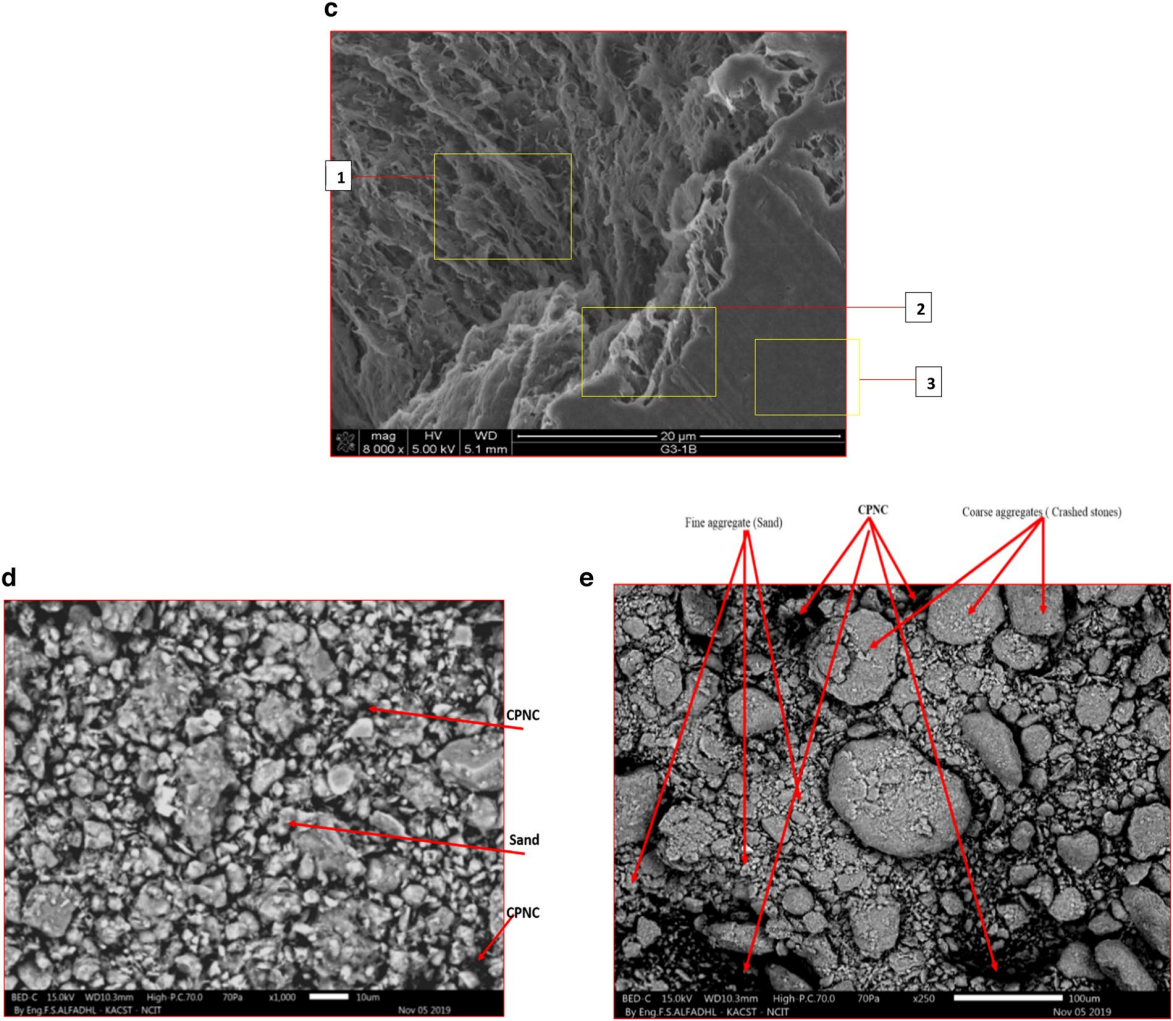
(**a**) SEM micrographs for MMT nanoclay layers before mixing with melted HDPE to produce CPNC. (**b1**) SEM micrograph of nonmelted pure HDPE M40060 particles, showing polymer particle shapes before melting or mixing with MMT to produce CPNC. (**b2**) SEM micrograph for melted pure HDPE. (**c**) SEM of CPNC (MMT and HDPE) showing the internal thickness and external nanostructure surface, homogeneity and nanoclay–polymer bond: (1) internal structure and fibre orientation, (2) thickness and layers, and (3) external surface of the sample. (**d**) SEM micrographs of CPNC mortar showing the homogenous microstructure of bonded sand and melted CPNC. (**e**) SEM micrographs of CPNC concrete showing the microstructure of bonded sand and aggregate with melted CPNC.

Characterizations results by SEM are shown for MMT, HDPE, CPNC, CPNC-concrete and CPNC-mortar where Fig. 5a, shows SEM micrographs for MMT nanoclay layers before mixing with melted HDPE to produce CPNC, while Fig. 5b1, shows SEM micrograph of nonmelted pure HDPE M40060 particles, showing polymer particle shapes before melting or mixing with MMT to produce CPNC. Figure 5b2, shows SEM micrograph for melted pure HDPE, and Fig. 5c, shows SEM of CPNC showing internal thickness and external nanostructure surface, homogeneity and nanoclay–polymer bond, where location (1) is internal structure and fibre orientation, location (2) is thickness and layers, and location (3) is external surface of sample. Figure 5d, is SEM micrographs of CPNC-mortar showing homogenous microstructure of bonded sand with melted CPNC, and Fig. 5e, is SEM micrographs of CPNC-concrete showing microstructure of bonded sand and aggregate to melted CPNC.

#### XRD

**Figure 6:**
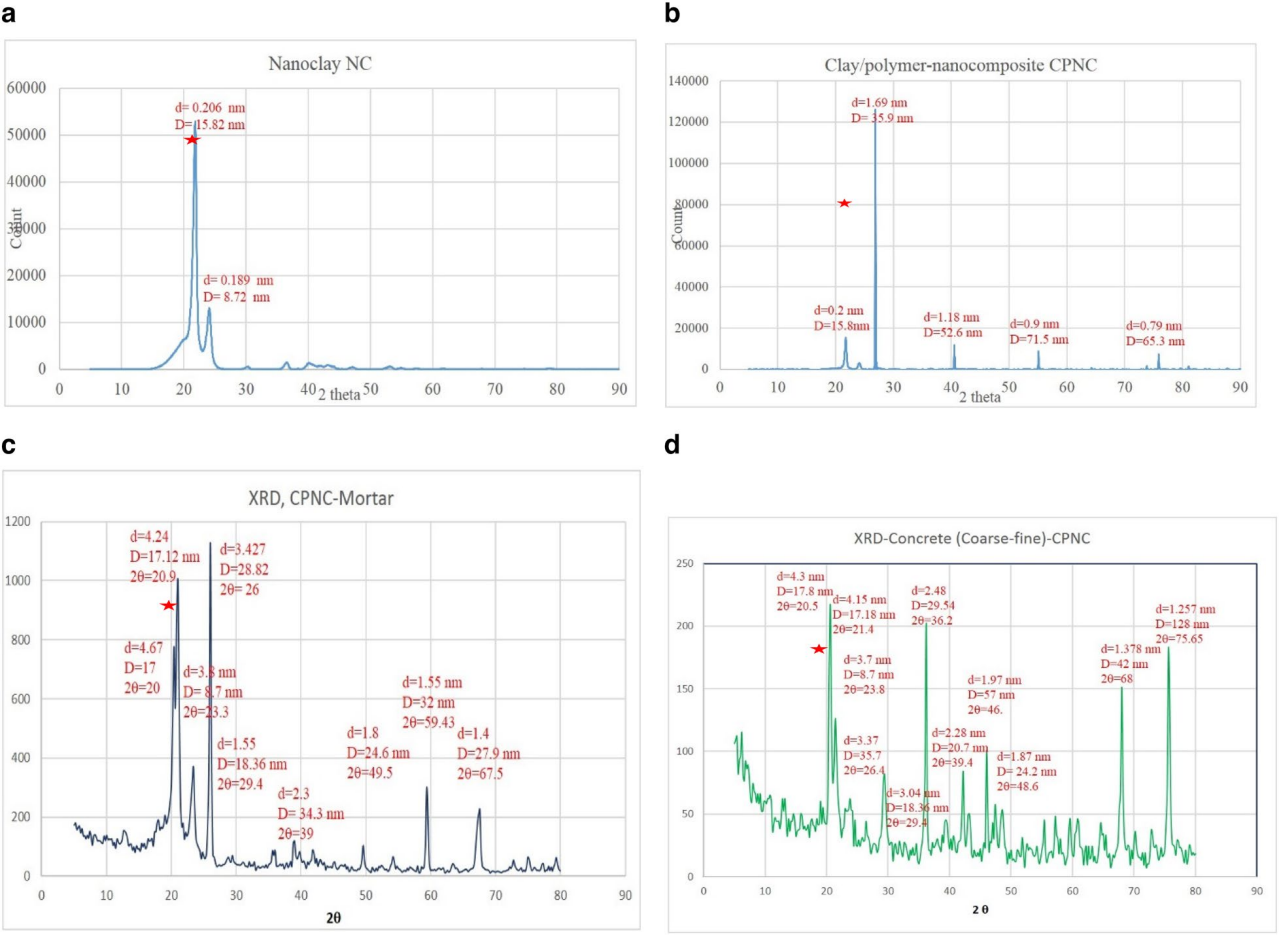
(**a**) XRD of MMT (I.30 T), showing original d between nanoclay layers (). (**b**) XRD result of CPNC (MMT mixed with melted HDPE), showing the change in d () between nanoclay layers due to mixing. (**c)** XRD results for CPNC mortar (melted CPNC mixed with sand), showing the change in d () between nanoclay layers due to mixing. (**d)** XRD results for CPNC concrete (melted CPNC with sand and coarse aggregate) showing the change in d () between nanoclay layers due to mixing.

Test results are shown in each of Fig. 6a, XRD of MMT (I.30T), showing original d between nanoclay layers (), Fig. 6b, XRD result of CPNC (MMT mixed with melted HDPE), showing change in d () between nanoclay layers due to mixing. Figure 6c, XRD results for CPNC-mortar (melted CPNC mixed with sand), showing change in d () between nanoclay layers due to mixing, and Fig. 6d, XRD results for CPNC-concrete (melted CPNC with sand and coarse aggregate) showing change in d () between nanoclay layers due to mixing.

## Discussion

### Mechanical properties

Regarding mechanical properties**,** based on ASTM, test of compressive strength is carried out for CPNC-mortar made of sand and MMT nanoclay/polymer nanocomposites where amounts of CPNC relative to sand are 5%, 10% and 15% of sand weight. Sand size passes through sieve #16; CPNC is made of HDPE and MMT with a ratio of 5% MMT nanoclay to HDPE. Table 3, shows test results of compressive strength of CPNC–mortar is 10.4 MPa) without additives; it has good strength relative to Portland-cement mortar, as shown in Tables 1, 2 and 3. Moreover, it is ductile mortar while Portland-cement mortar is purely brittle. CPNC-concrete made of crushed stones, sand and CPNC with different ratios (5%, 10%, and 15%) by weight. Sand particle size passes from sieve #16, crushed stone particle size passes from sieve #4, and CPNC is made of HDPE and MMT with a ratio of 5% MMT nanoclay and 95% HDPE. Table 2, is indicating that compressive strength equals 24.2 MPa, without any additives. It has good strength relative to known conventional Portland-cement concrete. Tested cylinders have diameters of 75 and 100 mm and heights of 150 and 200 mm. Load‒displacement curves are shown in Fig. 3a,b. Moreover, Table 2 and Fig. 2a2, show a comparison of mechanical properties, mechanical behaviours and failure modes of CPNC-concrete and Portland-cement concrete considering ACI^12^, BS^13^ codes and ASTM^14,15^ standards; there are large differences between newly developed materials and Portland-cement materials according to Fig. 2a2.

Regarding tensile strength test, which is a splitting test based on ASTM^14–17^, testing machine is a German-made Toni Technik GmbH, Model 2091 with a maximum capacity of 300 kN, as shown in Fig. 2a, while testing cylinder samples for compressive and tensile strengths, as shown in Fig. 2b,c, respectively. Test is carried out at room temperature. Test results of tensile strength are found based on ASTM C496-1^12–17^ standard for samples of diameter (D) = 10 cm and length (L) = 20 cm, as shown in Fig. 2a–c. Results are shown in Table 6, while tensile strength (T) is estimated based on Eq. (1).

1 $${\text{T }} = \, \left( {{\text{2P}}} \right)/\left( {{3}.{\text{14 LD}}} \right)$$

where P is maximum load at cylinder failure, L is cylinder length, and D is cylinder diameter. Average tensile strength is approximately 2.55 MPa, while average compressive strength of tested cylinders is approximately 25.0 MPa; these findings indicate that tensile strength is approximately 10.2% of compressive strength. According to ACI code, tensile strength of conventional Portland-cement concrete is approximately 6% of compressive strength. This phenomenon suggests that new CPNC-concrete has a tensile strength larger than that of conventional Portland-cement concrete. This result is due to ductility behaviour of CPNC-concrete. In addition, as shown in Fig. 2a–c, there are no failure modes apart from deformation, and there is no splitting of cylinders; in Portland-cement concrete, failure mode is usually splitting of cylinders to almost two halves through loading line. While for CPNC-concrete, there is no splitting, crushing, or sudden failure; there is only ductile deformation and small ductile cracks without brittle splitting or cracks. The deformation manifests as a very slight change in shape; this change is visible in diameter of cylinder, which becomes a parabola. There is a 5% change in diameter of cylinder from one axis, which becomes 9.5 cm instead of 10.0 cm; in other direction, it becomes 10.5 cm instead of 10 cm. Therefore, deformation changes cylinder diameter to a minor axis of 9.5 cm and a major axis of 10.5 cm, as shown in Fig. 2a–c. There is only deformation with very slight ductile longitudinal cracking at sides of cylinder, while conventional Portland-cement concrete behaviour undergoes sudden brittle failure at middle of cross section without ductile deformation. Ductile-deformation converts circular cross section of diameter equal D = 10 cm to an elliptical cross section with a vertical minor axis (a < D = 9–9.5 cm) and a horizontal major axis (b > D = 10.5–11 cm), as shown in Fig. 2c. Regarding comparisons, since new materials without Portland-cement or water manufactured with new thermal casting techniques have no previous similar materials, there are no similar data for comparison. Therefore, results are compared to Portland-cement concrete codes^12–17^. Comparison includes data in Tables 2, 3 and 4, Fig. 2a2 (failure modes) and Table 6.

**Table 6:** Comparison between CPNC concrete and Portland cement concrete.

Property	CPNC concrete	Conventional concrete
Material components	CPNC + aggregates	Portland cement + aggregates
Casting technique	Thermal melting in electric or microwave ovens	Wet mixing using water and Portland cement
Compressive strength	> 25 MPa	> 25 MPa
Tensile strength	More than 12% of compressive strength	6–10% of compressive strength (ACI code)^1–5^
Modulus of elasticity	Ductile; E = 106–285 MPa	Very brittle; E = 14–41 GPa
Specific weight	Light (γ = 1.5–1.8 ton/m^3^)	Heavy (γ = 2–2.5 ton/m^3^)
Fracture properties	Ductile fracture	Brittle fracture
Water absorption	Almost 0%	10% (ACI code)^1–5^
Steel corrosion	Almost no possibility	High possibility due to use of water
Hardening time	Final hardening 2–3 h	Final hardening 28 days
Construction time	Rapid (2–3 h)	28 days
Curing	No curing	28 days of curing
Water use	No	Large amount for casting and curing
Cracks	No shrinkage cracks	Shrinkage cracks due to processing
Ductility	Ductile (has large ductility due to CPNC)	Brittle material due to using Portland cement
Rapid processing	Rapid, needs just few hours	Needs long time (approximately 28 days)
Contamination	Possibility of contamination is low	Possibility of contamination is high
Homogeneity	Almost homogenous	Heterogeneous
Compaction	Easy self-compaction	Compaction needs mechanical vibrations
Crushing and sudden failure	There are no crushing, sudden cracks or splitting failures because it is ductile	Easy crushing, splitting and sudden failure with very rapid crack propagation
Moulds	Metallic with internal coating of aluminum sheets	Wooden or metallic forms
Advantages and disadvantages	Advantages: No water, no Portland cement, no pollution, light weight, no steel corrosion, rapid processing, ductile, no crushing, no rapid crack propagation	Disadvantages: Water use, contamination, heavy weight, steel corrosion, brittle cracks, crushing failure, rapid crack propagation

#### Fracture properties

Fracture properties results indicate fracture toughness and fracture energy, and they prove ductility of CPNC concrete; these results show that there is no sudden failure, crushing or rapid crack propagation of tested sample. Fracture properties, including fracture toughness (K_Ic_), fracture energy (G_f_), type of fracture and fracture mode, have been investigated for standard tests of notched beams under three-point loads, including pre-crack at bottom edge (single edge notch beam (SENB) tests). Results are shown in Table 4 and Fig. 4a,b. Fracture toughness^12–17,41,45^ or critical stress intensity factor K_Ic_, as shown in Eqs. (2) and (3), is a function of critical applied load (σ_c_), half notch length (a) and beam geometry and dimensions:

2 $${\text{K}}_{{{\text{Ic}}}} =\upsigma _{{\text{c}}} (\uppi {\text{a}})^{{0.{5}}} \left( {\text{F}} \right)$$

where F is a factor that depends on relative dimensions between cracks and location with respect to beam dimensions and geometry.

3 $${\text{F}} = \, \left[ {{1}.0{7 }\left( {{1 } + {3}.0{\text{3 a}}/{\text{b}}} \right)} \right]/\left[ {{2 }\left( {{3}.{\text{14 a}}/{\text{b}}} \right)^{{0.{5}}} \left( {{1} - \left( {{\text{a}}/{\text{b}}} \right)} \right)^{{{1}.{5}}} } \right]$$

where a is crack length and b is beam width. Fracture energy G_f_ is calculated based on ASTM^12–17,41^. Results are shown in Table 4 and Fig. 4a,b. It is recognized that (tensile strength) = 10.2% (compressive strength) for CPNC-concrete, while (tensile strength) = 6% (compressive strength) for Portland-cement concrete based on ACI code^12–17^. In addition, failure mode is ductile without splitting or brittle cracks. There is only deformation with very slight ductile longitudinal cracking at sides of cylinders; conventional Portland-cement concrete behaviour is sudden brittle failure at middle of cross section without ductile deformation. The ductile deformation converts cylinder circular cross-section diameter of D = 10 cm, to an elliptical cross-section with vertical minor axis of 9 cm and horizontal major axis of 11 cm, as shown in Fig. 2c. Fracture propagation proves that cracks are ductile where there is no brittle failure, crushing or sudden failure. Fracture test proves that thermally cast, no-cement concrete with no water for mixing or curing and rapidly developed strength has high energy due to its ductility; ductile materials always have large amount of energy while brittle materials always have small amount of energy which dissipate easily for brittle-failure. In other words, ductile materials usually have a large scale yielding and plasticity at crack tip before fracture initiation and crack propagation; brittle materials have small scale yielding and plasticity at crack tip before fracture initiation and propagation. Brittle materials always have low fracture toughness and fracture energy; ductile materials have high fracture toughness and fracture energy for same reason. Portland-cement concrete is always brittle with low fracture toughness and fracture energy, indicating that Portland-cement concrete always suffers from brittle fracture, rapid crack propagation and failure; CPNC-concrete is ductile material; it can resist fracture and sudden failure. Fracture properties are tested, as shown in Table 4 and Fig. 4a,b, where fracture energy G_f_ depends on fracture toughness K_Ic_, which depends on critical load, crack length, dimensions, and modulus of elasticity. Concrete structures have an essential demand for ductility to allow warning signs before cracking and failure to avoid sudden brittle failure. Therefore, recent CPNC-concrete is convenient for building construction since it has sufficient ductility. Results of K_Ic_ and cracking modes of tested samples under compressive and tensile strength show and prove ductility properties of CPNC-concrete. In addition, these cracks can be repaired easily by exposing cracked zone to local thermal effect to repair it without additional bonding materials. Fracture and crack propagation characteristics prove aim of developed CPNC-concrete where load‒displacement relationships exhibit elastic‒plastic shape, and deformation is ductile without sudden brittle failure or splitting, as shown in Fig. 3a,b which are different from known behaviour of conventional Portland-cement concrete^12–17^. Following basics of fracture mechanics science, ductile CPNC-concrete does not fail suddenly while brittle Portland-cement concrete fails suddenly, which does not have large deformation before failure; as in cases of ductile or brittle‒ductile materials.

#### Chemical composition

Regarding Chemical composition using EDAX characterization**,** EDAX analysis shows that NC is composed of Si, Al, and O, CPNC is composed of MMT nanoclay and HDPE polymer where MMT chemical elements are Si, Al, O and C, while HDPE contains C and O. Therefore, relative percentage ratios of CPNC chemical composition elements are 0.4, 0.23, 8.69, and 90.48 for Si, Al, O, and C, respectively. These ratios explain that MMT ratio is very small in comparison to HDPE, and verify concept of CPNC, which depends on reinforcing polymer matrix with exfoliated nanoclay platelets at a ratio of approximately 1–5%. EDAX analyses in Table 5 show elemental composition of no-cement concrete made of sand and CPNC, which includes O, Si, Al, Mg, Fe, K, Cu, Zn, Ca, Ti, Cl, and Si at percentage ratios of 51.4, 27.7, 8.7, 3.7, 3.2, 2.3, 1.1, 0.8, 0.8, 0.3, 0.1, and 0.1, respectively; CPNC-concrete made of sand, coarse aggregate and CPNC have compositions of 54% C, 28.6% O, 7.9% Ca, 7.8% Si, 1.2% Al, 0.4% Fe, 0.2% S, and 0.1% Cl. This finding proves homogeneity, well distribution, intercalation, dispersion, exfoliation, and bonding characteristics proving concept and properties of newly developed CPNC-concrete.

#### SEM characterization

Regarding SEM characterization, microstructure and morphology characteristics of MMT, HDPE , CPNC, CPNC-mortar and CPNC-concrete using scanning electron microscope (SEM) are investigated where Fig. 5a shows SEM micrographs for agglomerated MMT, Fig. 5b shows SEM micrographs for HDPE polymer pellets, Fig. 5c shows SEM micrographs of CPNC made of HDPE and 5% MMT, Fig. 5d shows SEM micrographs for homogenous microstructures of CPNC/sand, with very fine arrangements of bonded sand with melted CPNC instead of Portland-cement, and Fig. 5e shows SEM micrographs for CPNC-concrete components of melted CPNC made of MMT and HDPE mixed with sand and coarse aggregates, producing CPNC-concrete with microstructure of sand/CPNC/aggregate particles with CPNC bonding agent instead of Portland-cement. Figure 5a shows SEM micrographs for nanoclay; SEM micrograph in Fig. 5b shows microstructure of HDPE, and Fig. 5c shows SEM micrographs for CPNC. In addition, Fig. 5d shows SEM micrographs of CPNC-mortar, and Fig. 5e shows SEM micrographs for CPNC-concrete. The figures show microstructures of aggregate particles bonded to CPNC and distributions of NC particle reinforcements for CPNC-concrete. The interfacial bond is clear without any defects, pores, or cracks. The homogeneity of CPNC-concrete nanocomposite is clear. Some pre-cracks of aggregates are shown because coarse aggregate is made of large particles of basalt and dolomite that are crushed into smaller particles. The microstructure of CPNC-concrete shows homogeneity, bonding, morphology of CPNC showing NC fibres and polymer chains. Figure 5a shows morphology, size, and shape characteristics of NC particles. SEM micrographs prove concept and approach of developed no-cement concrete technique. The micrographs show no defects, no cracks, and good distributions of the particles with fine homogeneity and particle arrangement; this characteristic primarily contributes to the high strength of the concrete. The results prove that the no-cement concrete does not experience bleeding, segregation, non-homogeneity, defects or microcracks, unlike Portland-cement concrete. The results prove that the no-cement CPNC-concrete does not undergo brittle fracture or crack propagation since there are no internal defects, pores or cracks that may cause fracture or propagation under service loading, like Portland-cement concrete; this phenomenon occurs due to the concentrations of stresses around the defects, pores and microcracks, which may produce cracks under tensile, compressive or shear stresses. Therefore, CPNC-concrete is safe from crack initiation or propagation under service loads. CPNC-concrete microstructure proves good concrete self-compaction, self-arrangement, and self-distribution with good arrangement of aggregate particles in melted CPNC reinforced with MMT platelets or nanotubes like halloysite. The reinforced CPNC with high viscosity covers crushed-stones and sand particles producing high reinforced interfacial bonding after cooling and high strength, with ductile behaviour. CPNC-concrete does not have pores, cavities, or air bubbles due to thermal-casting technique and zero-water. SEM micrographs show microstructures of both CPNC-concrete and CPNC-mortar, indicating no-cracks.

#### XRD characterization

Regarding XRD characterization; Fig. 6 shows XRD results, which support understanding of distributions of particles and phases of MMT, HDPE, CPNC-mortar and CPNC-concrete, with information about changes in crystal sizes due to bonding and homogeneity characteristics. Figure 6a shows XRD result of MMT nanoclay powder, Fig. 6b shows XRD result of CPNC powder made of HDPE and MMT, Fig. 6c shows XRD result of CPNC-mortar, and Fig. 6d shows XRD result of CPNC-concrete. SEM micrographs accompanied by XRD for the no-cement CPNC-concrete show the microstructure of aggregate particles bonded to CPNC and distribution of NC particle reinforcement for CPNC-concrete. The interfacial bond and homogeneity of CPNC-concrete are noticeably clear. Figure 6 shows XRD chart of thermally cast no-cement concrete made of CPNC and sand, while XRD results indicate crystalline distribution of thermally cast no-cement concrete made of CPNC, coarse aggregate and sand with prediction and calculation of d and D relative to each other. Equation (4) represents Bragg's law^34^ for calculating d, while Eq. (5) represents Scherer’s law^35^ for calculating crystal size D.

4 $${\text{n}}\uplambda = {\text{2d sin}}\;\uptheta$$

5 $${\text{D }} = \, 0.{9}\uplambda /({\text{B cos}}\;\uptheta )$$

where λ (wavelength) = 0.15418 nm; n = 1.0; d is the spacing between atom layers; B is the peak width in radians, where B (radians) = (2 π) (B)/(360°), which is the peak width at half of the full peak height (FWHM); 2θ is the angle of diffraction; and D is the crystal size. The peak angle (2θ), distance between nanoclay layers (d), and particle size (D) for MMT, CPNC, CPNC-concrete and CPNC-mortar prove the exfoliation characteristics of nanoclay particles due to changes in the distances between layers (d), and they prove the bond between nanoclay particles and polymer chains through increase in particle size (D). In addition, these phenomena prove bonding between CPNC and sand, producing high-strength mortar through changes in both d and D. Moreover, these findings prove bond among CPNC, sand and coarse aggregates of crushed stones producing high-strength bonding for concrete. The results of XRD prove and match each of results of SEM and EDAX, verifying homogeneity, bonding, and fine arrangement of particles without cracks or defects. In addition, results of XRD prove results of mechanical and fracture property tests, where the bond produces high compressive and tensile strengths with good intrinsic parameters of fracture toughness and fracture energy, verifying ductility of the produced CPNC-concrete. As examples for the results of d and D shown in Fig. 6, which prove the changes in these parameters, at 2θ = 22˚, d = 0.206 nm and D = 15.8 nm; for the CPNC shown in Fig. 6, at 2θ = 22˚–26˚, d = 0.2–0.17 nm and D = 15.8–35.9 nm. For the zero-cement mortar nanocomposite shown in Fig. 6, at diffraction angles 2θ = 22˚–26˚, d = 4.24–3.3 nm and D = 17.2–28.8 nm; for CPNC concrete at (2θ) = 22˚–26^˚^, the changes are d = 4.3–3.7 nm and D = 17.8–35.7 nm. Figure 6 shows the XRD results. The change in distance (d) proves the good distribution and homogeneity of the nanoclay platelets, which represent the nano-reinforcement of the concrete composite. Additionally, it is essential to characterize the sizes of crystals of the elements in the no-cement concrete by using Sherrer's law^35^. The change in the crystal size proves the bond, which enhances the mechanical and fracture properties. The calculations of d and D depend on the values of angle θ and the peaks. The results of d and D, are estimated according to the Bragg equation^34^ and Sherrer's law^36^. The d distances of NC and CPNC change due to the exfoliation of the nanoclay layers and dispersion in the polymer matrix. Additionally, D is changed for NC and CPNC due to the processing of CPNC; both d and D almost do not change while processing thermally cast no-cement CPNC-concrete, verifying the stability of CPNC, which is the agent that produces the bond strength and other properties.

## Methods

### Fundamentals of the study concept and approach

This research is carried out experimentally to develop and investigate new concrete and mortar materials free of Portland-cement and water; Portland-cement is replaced by a newly developed material called nanoclay-based polymer nanocomposite CPNC without using water for mixing. It is known that common conventional construction materials, such as concrete and mortar depend on the use of Portland-cement; however, these industries are dangerous sources of environmental contamination causing severe human health diseases, such as cancer^1–11^. Therefore, this research involves developing a new green material for use instead of Portland-cement. Concrete is a heterogeneous, brittle, heavy material containing coarse and fine aggregates bonded by Portland-cement powder with water^12–17^. Conventional concrete is composed of 45–60% Portland-cement, and the amount of water is approximately 50% the amount of Portland-cement; this finding indicates that conventional concrete contains a large volume of Portland-cement and consumes a large volume of potable water for mixing and curing. Moreover, the mixing and casting of conventional concrete requires a long period (28 days) for processing and hardening. Conventional concrete processing consumes an additional large volume of potable water for essential curing during processing to prevent shrinkage cracks. This material is very brittle due to the use of Portland-cement^12–17^. Therefore, conventional Portland-cement concrete suffers easily from brittle fracture, rapid crack propagation and sudden failure; in addition, conventional Portland-cement concrete causes steel corrosion due to water use and absorption. Usually, concrete structures contain steel bars for reinforcing the concrete material to resist tensile stresses. Steel bars always suffer from exposure to corrosion processes, which causes severe deterioration of the steel material, reduction in the steel tensile strength, de-bonding between steel and concrete, crack formation and failure. The main reason for the corrosion process is the presence of water. Portland-cement concrete depends mainly on water for processing and curing, which indicates that it is impossible to prevent corrosion of steel bars inside the conventional concrete element. Additionally, this material suffers from its high thermal conductivity, representing a contamination source, heavy weight, water use, difficult processing, long hardening time, and the need for special techniques and precautions in hot and cold weather. Portland-cement is not eco-friendly, harming the environment, human health, and economy^1–11^. The Portland-cement industry causes substantial environmental pollution, and cement dust has dangerous effects on human beings; cement dust causes serious diseases, such as cement eczema, lung cancer, skin burns, and chromate allergies^1–11^. Special storage conditions are needed to avoid deterioration of mechanical properties due to temperature change and humidity^12–17^.

Some researchers have tried to enhance the properties of conventional Portland-cement^36–40^ by directly adding clay to Portland-cement. However, the present aim is different because previous researchers do not solve the problems of Portland-cement, but they encourage the use of Portland-cement and potable water. In this study, we aim to avoid Portland-cement use and reduce potable water use. Previous researchers have tried to add pure raw nanoclay particles to Portland-cement to produce conventional concrete mortar. The recommendations of concrete codes ACI^12^ and BS^13^ consider pure raw clay and silt as harmful substances that harm the mechanical properties of concrete causing debonding and cracks in concrete, making them prohibited materials in the concrete industry. Pure natural raw nanoclay particles are inorganic thermosetting hydrophilic materials in the form of sheets or nanotubes that absorb large amounts of water, causing harmful effects on concrete and steel if used as natural fillers; however, we convert them to another nanocomposite before using them in concrete. This study does not review the enhancement of Portland-cement mortar or concrete; instead, it aims to completely eliminate the use of Portland-cement. Some other researchers^31–33^ have looked for the enhancement of Portland-cement by using nanoclay; their research differs from code recommendations^12–17^, and it does not involve changing any negative effects of Portland-cement or replacing the material. For these reasons, raw nanoclay cannot be used in its natural shape as an inorganic agglomerated particle to enhance Portland-cement. Nanoclay particles should be covered with organic surfactant materials, such as octadecylamine, to modify particle surfaces into organic-like host materials to produce bonds between the coated clay particles and polymers, producing CPNC; CPNC includes nanoclay as a reinforcement for the polymer, which is a bonding agent, instead of Portland-cement. The processing method is a thermal casting process without using water for producing CPNC–concrete and CPNC–mortar; it avoids problems with Portland-cement while achieving new important properties. The developed material overcomes the problems of conventional concrete, which depends on a green natural nanocomposite-clay-based polymer nanocomposite (CPNC)-composed of a small ratio of exfoliated nanoclay of approximately 5% NC mixed with 95% high-density polyethylene (HDPE) processed through an extruder by melting synthesis. This research represents one of the new applications of nanocomposites made of natural green nanoclay particles and polymers; nanoclay platelets are used as reinforcement fillers to change and enhance the properties of the polymer matrix. Due to the unique properties of natural nanoclay platelets, such as very large surface areas and other thermal and mechanical properties, they enhance the thermal, mechanical, and fracture properties of polymers, transforming the polymers into sustainable nanocomposites resisting degradation, retarding fire, resisting heat, enhancing tensile and compressive strengths, and changing the mechanical behaviour and failure modes. Due to previous studies from us and of other researchers^18–30,42–44^, it is found that the best ratio of MMT to enhance the polymer properties producing CPNC is 5%. Researchers studying CPNC made of MMT nanoclay and polymers have usually tried to use several ratios between 1% MMT, 3% MMT, 5% MMT, and 10% MMT nanoclay, where they prove that 5% nanoclay is the best; if the amount of nanoclay exceeds 5%, it may enhance thermal properties but deteriorate the material mechanical properties. Therefore, we use a ratio of 5% CPNC to obtain good results and avoid concrete deterioration simultaneously. However, the idea of the research is new, and there are no similar CPNC-concrete or CPNC-mortar materials with zero Portland-cement or zero water made by the thermal casting technique utilized in our current study or by any other technique. Our study involves newly developed materials with new techniques. Therefore, there are no previous information or data regarding the mix design of the CPNC ratio^18–30,42–44^. Therefore, we use three percentage ratios of 5%, 10% and 15% CPNC to investigate whether the ratio of 15% represents the best ratio. The results indicate that the best global ratio of nanoclay particles embedded in the CPNC-concrete is (5% MMT)*(15% CPNC) = 0.75% MMT nanoclay. When using 10% CPNC, the MMT ratio is 0.5% of the total volume of concrete; for 5% CPNC, the MMT ratio is 0.25% of the concrete volume. Therefore, we cannot increase the ratio to more than 15% CPNC to avoid deterioration of the concrete; the other ratios of 5% and 10% have good results but are still not better than the 15% ratio. MMT nanoclay is expensive, and CPNC-concrete is inexpensive relative to the benefits. However, conventional Portland-cement design codes, such as ACI^12^, consider pure raw clay particles to be harmful substances for concrete; the harmful effects are increased by the 1% ratio. Although our nanoclay is not pure raw, it is still in the range of the allowable ratio of concrete design codes. In addition, since our new material is different from Portland-cement concrete for all sides, it needs special design codes. CPNC is a fine powder with special mechanical and thermal properties that enhance concrete and mortar properties. CPNC is a ductile material that reduces cracking, is thermally resistant, has good mechanical properties, and increases toughness and fracture energy. In this research, we develop CPNC–mortar and CPNC–concrete without Portland-cement or water using small dry ratios (5%, 10%, and 15%) of CPNC mixed with sand or sand and crushed stone, respectively. After dry mixing in special moulds, thermal melting synthesis is applied in electric ovens at 220–250 °C. The produced thermal casting CPNC-concrete is a homogenous, lightweight, low-density, self-compact, and ductile material without pollution. This material is a rapidly processed concrete without fracture, crushing or sudden failure. The study includes material synthesis, mechanical and fracture property testing, and characterizations of chemical components, microstructures and crystallography using SEM, EDAX and XRD. The results are suitable for an eco-friendly construction industry. Using CPNC as a bonding agent instead of Portland-cement reduces environmental pollution and potable water use, where CPNC-concrete contains 5–15% CPNC to produce CPNC-concrete within approximately three hours. Portland-cement concrete is not thermally resistant; CPNC–concrete is suitable for building insulation because it contains CPNC, which is thermally resistant.

The concept of the research is to produce green concrete that is suitable for construction by reducing the problems of conventional Portland-cement concrete, as shown in Fig. 1e,f. In conventional concrete, Portland-cement is the main material acting as a bonding agent that is responsible for creating interfacial bonds between aggregates to produce concrete strengths; it connects all particles to each other, producing a continuous solid material that handles mainly compressive stresses^12–18^. While this material exhibits other properties, such as tensile strength and shear strength, Portland-cement is a very dangerous material^1–11^. The Portland-cement industry produces high-levels of CO_2_ pollution and consumes a large amount of oil and gas, additionally generating substantial amounts of cement dust and harming humans, animals, and plants. Moreover, Portland-cement is very easily affected by water vapour or humidity, and it requires special methods and buildings for storage. Portland-cement needs a lot of water for mixing with aggregates to produce enough bonding; it takes a long time and requires a special curing process involving water for almost one month. Hot climates or cold climates need very special techniques and durations for mixing and hardening. The material resists brittle fracture, sudden failure and crushing because of its ductility. The concrete undergoes fast processing, developing strength rapidly in a few hours. The material does not need water curing as in the case of conventional Portland-cement. The concrete may consist of coarse aggregate (e.g., gravel, dolomite, or basalt), fine aggregate (sand) and CPNC instead of Portland-cement. Additionally, this material can be made of only fine aggregate and a nanocomposite-based mortar. The ratio of coarse aggregate to fine aggregate is 2:1, but it may change according to the required material properties, such as strength. The nanocomposite is a CPNC that consists of a polymer matrix reinforced with nanoplatelets of MMT montmorillonite nanoclay or nanotubes, such as halloysite. The ratio of the CPNC to the aggregate changes between 5, 10, and 15% according to the weight, which controls the strength of the concrete. The CPNC ratio controls the density, porosity and specific weight of the concrete. For mix design, fine aggregate, and dry powder CPNC should be mixed first until homogeneity for approximately 5–10 min; then, it is mixed with coarse aggregates for approximately 5–10 min until it is homogenous. In this study, we include the steps of material preparation, moulding, mixing, casting, heat processing in ovens, cooling, removing the moulds, preparing the samples for testing, testing (cubes and cylinders for compressive strength and beams for mechanical and fracture properties)**,** and characterization using XRD, EDX and SEM, analysis of the results, discussion, and conclusion**.** CPNC is prepared first by using a twin screw extruder and pelletizer to produce a nanoclay–polymer nanocomposite (CPNC) powder reinforced by nanoclay platelets or nanotubes. CPNC dry powder is mixed with aggregates covering all aggregate particles; then it is melted by heating, melting all aggregate particles, and generating bond strength. After cooling, a high interfacial bond is achieved without fractures or cracks, producing highly ductile, self-compact, no-cement, and no-water CPNC concrete. The temperature is not less than 220 °C. The temperature and heating time control the strength of the concrete; the temperature must not be less than the melting point of the CPNC. The concrete is compacted by self-compaction under the weight of the aggregates. The microstructure and particle arrangement depend on the temperature and CPNC ratio. The casting method does not allow the possibilities of cracking or fracture. The density of CPNC concrete is 1.5–1.8 g/cm^3^, while the density of conventional Portland-cement concrete is 2.2–2.5 g/cm^3^. The aim of manufacturing the newly developed concrete is to produce green ductile concrete with functional properties, such as ductility, eco-friendliness, light weight, fracture resistance, thermal resistance, crushing resistance, corrosion resistance, self-compaction, high homogeneity, rapid hardening, high strength, reduced pollution, sudden failure resistance, without Portland-cement or curing and through a dry mixing process. This material can be applied for all construction and structural purposes, especially in arid and semiarid areas. The concrete requires a special type of casting. CPNC is reinforced with NC. Figure 1 indicates the steps of the concrete nanocomposite processing. No-cement no-water concrete consists of green CPNC and natural aggregates, where clay/polymer nanocomposite CPNC is made of HDPE and NC nanoclay with a 5% NC ratio of MMT; the fine aggregates (sand), passing pass sieve #16 and the coarse aggregates (crushed stones) pass through sieve #4 based on ASTM. Regarding both fine and coarse aggregates, sand is washed to a particle of size less than 1.18 mm and a density of 2500 kg/m^3^; the coarse aggregate is washed crushed stone with a particle size less than 4.75 mm and a density equal to 2570 kg/m^3^. However, the Portland-cement density is 2250 kg/m^3^, while the nanoclay density is 260 kg/m^3^ and the HDPE density is 980 kg/m^3^. The density differences between nanoclay and polymer relative to Portland-cement and aggregates are clear.

Regarding testing and characterizations, testing includes mechanical and fracture properties, while characterization includes microstructure, interfacial bond, homogeneity, particle distribution using SEM, chemical composition using EDAX and crystallography, and changes in particle dimensions and distributions by XRD. The newly developed material is a thermally cast, no-cement, no-water, low-density, self-compact, ductile, corrosion resistant, brittle fracture resistant, functional, and green nanocomposite concrete. A new concrete material is fundamentally developed, tested and characterized. This material is made of aggregates and nanocomposite materials without cement or water. The material resists fracture, sudden failure and crushing. The casting technique depends on new technology using thermal casting through heating by microwaves or normal ovens or mixing under heating by thermal mixers. The concrete is quickly processed in a few hours to have a high strength; the processing time depends on the size and required type of concrete. This material does not require curing; it resists and retards fire. This CPNC-concrete can resist dynamic loads since it is ductile. The material has a high fracture toughness, fracture energy and strength without additional additives; it is thermally resistant, and controls heat transfer due to the use of thermally resistant CPNC. This material consists of aggregates and nanocomposites. The aggregate may consist of coarse (e.g., gravel, dolomite, or basalt) and fine (sand) aggregates. Additionally, the aggregate can be made of only coarse aggregate and nanocomposite (no fine aggregate) or only fine aggregate and nanocomposite (no coarse aggregate). The ratio of coarse aggregate to fine aggregate is 2:1, but it may be changed according to the required strengths and properties. The nanocomposite is a clay-based polymer nanocomposite CPNC that consists of a polymer matrix reinforced with nanoplatelets of MMT nanoclay or nanotubes. The ratio of the CPNC to the aggregate changes to 5%, 10%, and 15%, depending on the weight. The CPNC ratio controls the strength of the concrete. The CPNC ratio controls the density, porosity, and specific weight of the concrete. For mix design, fine aggregate, and dry powder CPNC should first be mixed until homogeneity for approximately 5–10 min and then mixed until homogeneity with coarse aggregates for approximately 5–10 min. The CPNC may be mixed in a hot mixer under various temperatures, depending on the melting point of the CPNC; the melting point changes based on the type of polymeric nanoclay composite. Then, the CPNC is cast in suitable moulds, where it is cooled normally. After a few hours, it is cooled and achieves the required strength without the need for curing. The coated moulds by aluminium, plastic or CPNC sheets are removed. Another method of casting involves casting the dry concrete mix in the moulds and microwave or oven heating the moulds for separate precast concrete elements. The temperature is at least 220 °C. The temperature and heating time control the strength of the concrete. The concrete is compacted by self-compaction under the weight of the aggregates. The microstructure and particle arrangement depend on the temperature and CPNC ratio. The Portland-cement industry produces a ratio of contamination of approximately 8% of CO_2_ pollution, which is a high ratio due to the industry being a single industrial product^1–11^. As basic data based on worldwide codes, concrete materials contain aggregates, Portland-cement, and water^12–18^. Aggregates of sand, gravel and crushed stones should always be clean particles and free of harmful substances^12–15^. Portland-cement under good storage conditions is a dry fine powder. Clean potable water is essential for mixing with cement^12–15^. The design of the concrete mix contains a cement content ratio equal to (0.35–0.6) with respect to the total unit volume of 1.0 m^3^. The ratio of cement content depends on the required compressive strength of concrete, where increasing the cement content ratio will increase compressive strength and vice versa. Moreover, the water content is a ratio of the cement content, where the average water content ratio is approximately (0.35–0.60) with respect to cement content. Water is essential not only for cement hydration but also for concrete curing, which is potable or distilled water^12–15^. This volume of water is very large with respect to the required volume of concrete for the construction of any structure, such as a building, bridge or dam. Water specifications are very restricted for containing any undesired substance that may harm the cement bond to aggregates, compressive strength, and cause steel corrosion^12–15^. More water is still needed for concrete curing^12–15^ for obtaining the maximum compressive strength after 28 days of casting. On the other hand, water is very rare in arid and semiarid areas, while underground water and sea water are not allowed for the concrete industry because of the presence of high ratios of chlorides and sulfates^12–15^. Therefore, the current research aims to save water in addition to avoiding Portland-cement contamination.

### Materials

Materials include montmorillonite (MMT) nanoclay (NC), high density polyethylene (HDPE) for producing clay-based polymer nanocomposites (CPNC) and aggregates for producing mortar and concrete by using CPNC instead of Portland-cement. Aggregates are fine and coarse; the fine aggregate is washed sand of sizes less than 1.18 mm with densities of 2500 kg/m^3^, passing through sieve #16; coarse aggregate is crushed stone of sizes less than 4.75 mm with densities of 2600 kg/m^3^, passing through sieve #4. Sand is used alone for mortar; for concrete, sand and crushed stones are used with a sand: crushed stone ratio of 1:2. The properties of montmorillonite nanoclay (MMT I.30T), HDPE (M40060) and CPNC are shown in Table 1. The raw materials are shown in Fig. 1a for MMT, HDPE, CPNC, CPNC-mortar and CPNC-concrete. The particle size of each raw material of MMT nanoclay, HDPE, and CPNC is tested using a laser particle analyser by wet analysis; the results are shown in Fig. 1b, where X (mm) is the particle size and Q3 (X %) is the particle size percentage in the material. The main processing equipment is the extruding system for producing CPNC, as shown in Fig. 1c; the moulds and electric ovens for the thermal casting of CPNC-mortar and CPNC-concrete samples of cylinders, cubes and beams are shown in Fig. 1d. The synthesis procedures of CPNC, CPNC-concrete and CPNC-mortar are summarized in the flowchart shown in Fig. 1e; Fig. 1f shows a sketch of mixing the sand and CPNC particles, which are HDPE particles reinforced with MMT platelets.

### Instruments and specimens

The instruments include a computerized extruding and mixing system for producing CPNC powder, including a laboratory station, twin screw extruder model (L/D = 40, L is screw length, D is screw diameter), water bath, HDPE feeder, NC feeder and high-speed pelletizer. Other instruments are used to produce no-cement and no-water concrete, including mixers, ovens, and standard moulds for cylinders, cubes, and prisms. There is a testing machine for mechanical and fracture toughness testing, including a universal displacement control machine, for testing specimens of cubes and cylinders for compressive and tensile strengths and for testing the fracture properties of beams. The characterization instruments include a scanning electron microscope for SEM and EDAX and an X-ray machine. Testing samples include standard cylinders of dimensions (75 mm diameter and 150 mm height or 100 mm diameter and 200 mm height), standard cubes to mortar test dimensions (50*50*50 mm) and beams for testing fracture properties. The experimental work has three main phases: the first phase includes producing the CPNC, the second phase includes producing zero-cement CPNC-mortar, and the third phase includes producing zero-cement CPNC-concrete. Each phase includes the testing of mechanical properties and fracture properties in addition to EDAX, SEM, and XRD characterization processes, as shown in the associated tables and figures. Mechanical and fracture properties are determined in KSU laboratories at room temperature (21 °C) using a universal testing displacement control machine at a displacement rate of 0.2 mm/min. The components of newly developed clay-based nanocomposite concrete contain aggregates of fine particles, coarse particles, or both with CPNC. CPNC produces bonds between aggregate particles without Portland-cement or water on any other chemical additives. CPNC is a green material with good mechanical and fracture properties in powder, pellet, thread, rod, or sheet shapes. CPNC is a dry powder for producing CPNC-concrete. Additionally, the material can be used as pellets for producing concrete nanocomposites; however, fine powder is very important for homogeneity purposes. CPNC is added to the aggregate at a ratio of 5%, 10%, and 15% by weight; the mechanical and fracture properties and self-compaction ratio depend on the CPNC ratio in the concrete mix. Increasing the CPNC ratio increases the self-compaction ratio and the density. Therefore, the density, compressive strength, mechanical properties and fracture properties are controllable. The most important characteristic of CPNC concrete is ductile behaviour. Special mould preparation coted internally with thin sheets of aluminium is needed to facilitate sample removal from the moulds. Figure 2a–c show the test setup during sample testing; Figs. 3 and 4 represent load–displacement relationships showing the clear ductility behaviour of CPNC-concrete. Tables 1, 2 and 3 present summaries of the results of the testing of HDPE, CPNC, CPNC-mortar and CPNC-concrete. Figure 5a indicates the microstructure, morphology, and composition of the new concrete nanocomposite showing all components, including CPNC platelets and clear bonds between CPNC and aggregates. The required temperatures for the tested cases are in the range of 220–250 °C for a casting time of approximately 120 min. Nanoclay NC platelets in the composite are responsible for reinforcing the CPNC, which is the bonding agent. Therefore, NC particles strengthen the polymer matrix, resist fracture and degradation, and enhance the mechanical and fracture properties of the newly developed CPNC concrete. The number of specimens for each experimental test is 15. The results represent the average value for each test with a calculation of the standard deviation for each test where the errors are too small and the results are almost identical, as shown in the Tables 2, 3 and 4. The standard deviation (σ) is calculated based on Eq. (6):

6 $$\upsigma = \, [ \, \left( {{1}/{\text{N}}} \right) \, (\Sigma _{{{\text{i}} = {1} - {\text{N}}}} ({\text{x}}_{{\text{i}}} -\upmu )^{{2}} ]^{{0.{5}}}$$

where x_i_ is individual sample result, μ is mean value, and N is total number of samples.

### Synthesizing technique for CPNC, CPNC concrete and CPNC mortar

#### CPNC

The first phase of experimental work is conducted to develop a CPNC bonding agent instead of Portland-cement. The agent consists of 95% pure HDPE M40060, reinforced by 5% MMT NC nanomer (I.30T) synthesized by melt processing using a well-computerized and well-controlled high shear twin screw extruding system. CPNC is produced in a powder shape to be mixed with sand and crushed stones. The properties of the MMT nanoclay, HDPE polymer and CPNC are shown in Table 1. Figure 5a,b1,b2,c show SEM micrographs of MMT nanoclay, HDPE M40060, and CPNC, respectively. Regarding the SEM micrograph of CPNC, that micrograph is understood clearly if we show or imagine it for a melted pure polymer matrix without nanoclay platelets or extrusion. In that case, there is only a homogenous material without dispersed exfoliated nanoclay particles. Additionally, when mixing nanoclay with polymer manually without extrusion, the nanoclay does not have a single orientation; however, it is similar to a hybrid material with a nonuniform or homogenous orientation or distribution. In our research, the nanoclay composite is homogenously oriented. The orientation is in the direction of extrusion of moving twin screw extruders. Using SEM to characterize polymers and polymer-nanocomposites is very difficult and requires extensive experience handling microscopes and performing characterizations since the electron ion beams of the microscopes easily melt the sample due to their high temperatures, changing the sample microstructure and producing debonding between the dispersed nanoclay platelets. In hard solid or otherwise plastic materials, the ion beam can crack the samples or cause electrical charging, producing untrue results. Therefore, the characterization of clay/polymer nanocomposites and plastic materials is very difficult to carry out. Therefore, the SEM micrograph shows nanoclay fibres and the orientation of clay fibres and easily shows material thicknesses in some zones due to the arrangement of nanoclay layers; some parts are smooth during manufacturing, hiding the nanoclay fibres, as shown in Fig. 5c. Figure 5b shows the dry particles of pure HDPE; melted pure HDPE without adding to or mixing MMT nanoclay is shown in Fig. 5b2. These microstructure morphologies are different from those shown in Fig. 5c due to presence of MMT. MMT nanoclay nanomer (I.30T) is surface modified using the organic surfactant oxtadecylamine to facilitate the bonding of MMT nanoclay and HDPE polymer to enhance mechanical and fracture properties by producing a new nanocomposite CPNC. Bonding between nanoclay particles and polymer systems is the main factor producing nanocomposites since any debonding defect at the interface between the two materials produces cracks. The properties of MMT (I.30T) are shown in Table 1. The CPNC-synthesizing technique is called the melting processing technique^18–30^, and it is based on the melting point of HDPE. The system extrudes and produces CPNC as a raw powder material by adding dry NC powder to dry HDPE pellets in a twin screw extruder under the thermal effect of 250 °C; the powder undergoes a high shear extruding process to exfoliate and disperse MMT particles in the polymer chains. Before and during this process, the vacuum works to prevent any possibility of producing voids, gases, air bulbs or defects in the product. Then, the powder goes to the exit zone (die) to produce rods or threads. Then, the material passes through a cold-water basin at a suitable speed for cooling the rods. Then, the material passes inside a granulator with speed control to convert the rods into powders or pellets and collect them in a container. This process produces the required CPNC powder; additionally, it can be repeated several times to produce the required CPNC. Then, CPNC powder is added to the aggregates to start the processing of non-Portland-cement concrete. Both the mechanical and fracture properties of CPNC are investigated based on the standards of ASTM (D 638)^16^ and ASTM (D 5045)^17^. The results are shown in Table 1. Figure 3a indicates the stress‒strain relationships under tension for both pure HDPE M40060 and CPNC made of 5% MMT NC 1.30T and 95% HDPE M40060; the figure shows a clear and large difference in the behaviour and an increase in the stress capacity of CPNC. Additionally, Fig. 4a shows the change and increase in the stress capacity and behaviour of CPNC relative to pure HDPE M40060 during fracture toughness testing. The results show that nanoclay can change the mechanical and fracture behaviours by increasing the capacity of the nanocomposite. Then, the nanoclay is added to the sand and crushed stone as a bonding agent to replace Portland-cement while producing CPNC concrete.

### CPNC concrete and CPNC-mortar

The materials are shown in Table 1, where CPNC mortar consists of just two materials: sand and CPNC. The sand is natural washed with a maximum dimension of 1.18 mm passing through sieve number #16 according to standard specifications of ASTM^12–18^. The CPNC powder is produced in our nanoclay laboratory through a melt processing technique by using a commercial surface-modified MMT nanoclay: I.30T nanomer. CPNC mortar is synthesized by thermal casting and melting processing using electric ovens and cubic moulds of 5 × 5 × 5 cm^3^ and beams of 4 × 4 × 16 cm^3^ coated internally with thin aluminium sheets**.** CPNC concrete consists of CPNC powder and a mix of sand and crushed stones with a sand/crushed stone ratio of 1:2. CPNC–concrete is synthesized by a melt processing technique. The tested samples are cylinders that are tested for compressive and tensile strengths with standard dimensions of diameters of 7.5 and 10 cm and heights of 15 and 20 cm; the cylinder moulds are internally coated with thin aluminium sheets to facilitate sample removal. Other beam samples of standard dimensions (4 × 4 × 16 cm) made in internally coated moulds with thin aluminium sheets are prepared for fracture property testing. All samples are synthesized thermally at 250 °C using electric ovens. The first step of processing is mixing 5% CPNC dry powder with 95% dry washed sand for 5–10 min through a high shear mixer. The time of mixing is an important variable for a good homogeneous distribution of the composite for conventional concrete made of Portland-cement or for any composite based on Portland-cement and water; in our research, time is a very important difference between our newly developed material and conventional Portland-cement composites. This important difference occurs for several reasons. We are not using raw nanoclay or Portland-cement. Our materials include sand, crushed stones and CPNC powder. All of the sand, crushed stones and CPNC powder materials are dry, hydrophobic and cohesionless particles that are not sticky and can easily be distributed and mixed. Nanoclay particles in our material are not added to sand and crushed stone as natural raw nanoclay; instead, they are already embedded in the polymer matrix to be applied as cohesionless CPNC particles. Therefore, there is no cohesion between particles during mixing, which may prevent homogenous distribution during the mixing process without the need for a long duration; instead, the material requires a very short mixing time. For Portland-cement composites and natural nanoclay, both agglomerated particles and cohesive material absorb water easily from the surrounding atmosphere because both are hydrophilic. This hydrophilicity makes mixing natural nanoclay or Portland-cement with other materials very difficult and nonhomogeneous; this characteristic indicates that the materials require water to facilitate mixing and distribution. Therefore, the uniformity levels of the distributions of Portland-cement or pure natural nanoclay to any other material are very difficult and may be impossible to be homogenous and uniform; in our case, it is very easy to mix the materials with very high uniformity and homogeneity, as seen in the cross sections of the cylinders in the manuscript. Moreover, nanoclay particles are always agglomerated at the microscale; this agglomeration is impossible at the nanoscale if the particles are directly mixed with Portland-cement or any other material, such as sand or crushed stones, even if water is used in the mix. Nanoclay can only be at the nanoscale if the agglomerated nanoclay particles are exposed to the effects of high shear, such as when using a powerful twin screw extruder, as in our study, or when using similar devices, such as tows and three roll mills, several times. Otherwise, the MMT clay particles still agglomerate at the microscale or larger, which does not produce nanocomposites. In addition, nanoclay requires a very large amount of water and a very long time to mix until it is on the nanoscale. This aspect is the actual challenge, and its study has generated competition between researchers and companies working in the field of clay-based nanocomposites, especially for MMT nanoclay; mixing may be easier for clay nanotubes, such as halloysite nanoclay, due to the high bonding between layers of MMT nanoclay. The only difficulty in the mixing process is during the manufacturing of CPNC using the extruding system. Then, the admixture is installed in cylinders, cubes and beams that are already internally coated with thin aluminium sheets to facilitate specimen removal after synthesis. Then, cylinders, cubes and beams are installed in the oven at 220–250 °C. It is better to turn the oven on before installing the samples until the temperature increases to the required 220–250 °C. The samples are left in the oven for approximately 30 min for the small cubes, and this process continues for other cylinder and beam samples for approximately three hours. As a precaution for compaction, the mould height must be increased for each cylinder by an additional 2.0 cm and for each cube and beam by an additional 1.0 cm; the mould height increase is achieved by increasing the height of the aluminium sheets because during melting, the CPNC concrete and CPNC mortar are self-compacted, reducing the height of the samples by a small amount. The same procedures are made for the beams (prisms) for mechanical and fracture property testing. For the beam samples undergoing fracture property testing, small notches with maximum lengths of approximately 2.0 cm are made at the middle of the bottoms of the beams for testing under the 3-point load SNB based on the ASTM standard^14–17^; other samples are tested under the same conditions of three-point loads without notches to investigate the maximum critical load of the tested beams of zero-Portland-cement. The results are shown in Tables 1, 2, 3 and 4 and Figs. 1, 2, 3 and 4. Synthesis processes of both CPNC concrete and CPNC mortar are developed through steps that start with mixing the dry particles to a homogenous form; CPNC powder is first mixed with sand, then added to coarse aggregate and mixed for approximately 5–10 min. Then, different moulds are prepared by lining them with thin layers of aluminium sheets. Next, the dry mix is poured in the mould slowly with or without initial compaction. Afterwards, the mould is installed in a suitable oven (microwave or normal heater oven) or exposed to heating sours at the required temperature and left for the required time. Then, the oven is turned off and the moulds are left until they cool to atmospheric temperature. After that, the moulds are removed. Then, dry mixing is performed using a hot mixer with a high temperature and then poured in the moulds and left for cooling. Cooling may be carried out using any suitable cooling system or material for rapid cooling according to the required production properties. For dry mixing and dry pouring then heating, there are voids between the particles of aggregates and CPNC. While melting CPNC particles to cover the aggregate and fill the voids with interfacial bonding between the aggregates, the aggregate particles move down by gravity through the melted CPNC, filling the voids with a fine arrangement and producing dense concrete almost without voids created through a strong bond; the high-strength concrete nanocomposites are reinforced with nanoclay. The melted CPNC helps the aggregate particles settle, producing self-compaction without any external effects or forces. This process produces self-compact concrete specimens with high compressive strengths and no voids. The method is actually a sintering technique without any external pressure. The absence of voids prevents the creation of stress concentration zones, microfractures and crack propagation; this phenomenon indicates that material is produced with excellent fracture resistance properties. The new concrete nanocomposite is produced to cast any structural element with steel reinforcement on site or as a precast element. Regarding the synthesis technique, the materials consist of coarse and fine aggregates, with only one of them representing the main material of the composite matrix; the particles are bonded to each other by bonding a high functional material, which is the CPNC clay/polymer nanocomposite. Clay polymer nanocomposites are highly functional materials made of polymers reinforced with green nanoclay platelets or nanoclay nanotubes. The CPNC has new superior properties, including physical, chemical, mechanical and fracture properties. CPNC is a ductile, brittle, or semiductile/semibrittle material mixed with dry powder aggregate, producing a very fine homogenous composite. Then, CPNC is poured into a suitable metal mould based on the required structural element. Before pouring the composite mixture, the mixture contents are designed based on the required concrete strength; the moulds are coated internally using a very thin thermal lining or coating layer to prevent bonding between the mould sides and the concrete. The composite mix is exposed to a suitable temperature degree through microwave heating or normal heating by any reasonable technique, such as ovens, to produce precast elements or through thermal mixers to produce melted composites ready for casting. The moulds are metallic. The heating continues for a certain time based on the required strength and microstructure characteristics and the dimensions of the structural elements of the concrete composite. Then, heating is stopped to cool the concrete from the surrounding atmosphere or by the cooling technique. Cooling by the surrounding atmosphere may take several hours. The concrete nanocomposite starts hardening directly when the cooling period starts. Fine homogeneity is achieved when the particle sizes are almost equal and small, as in the case of sand; very fine aggregates can be mixed easily with CPNC dry powder, producing very fine homogeneity, as shown in the surface area in Table 1. The CPNC ratios to the aggregate are 5%, 10% and 15% by weight. The best results are for the 15% ratio. The compressive and tensile strengths and the compaction characteristics depend on the CPNC ratio; the changing ratios of volume due to compaction are 5%, 10%, and 15% for CPNC ratios of 5%, 10%, and 15%, respectively. The deformation and cracking modes of the tested samples are ductile without brittle cracks. There are no shrinkage cracks. It is lightweight concrete with a density of 1.5–1.8 g/cm^3^, which is suitable for applications in all fields of civil and construction engineering.

## Testing and characterization

### Mechanical and fracture properties testing techniques

The mechanical and fracture properties and the water absorption characteristics of CPNC mortar and CPNC concrete samples are tested based on ASTM^14–17^. The tested specimens are cylinders, beams, and cubes. The cylinders are tested under compressive loads based on the ASTM standard test to investigate the compressive and tensile strengths of CPNC concrete; cubes are tested to check the compressive strength of CPNC mortar. Additionally, the material ductility, stress‒strain relationship and modulus of elasticity are evaluated. The beams (standard prisms) of both CPNC concrete and CPNC–mortar are tested to check the fracture properties, including fracture toughness and energy. In addition, a water absorption test is carried out based on ASTM, showing that the CPNC mortar does not absorb any considerable water amount after measuring the cube weight before and after immersing the cubes in water at room temperature. The tested samples and results are shown in Fig. 2a–c and Tables 1, 2 and 3. A detailed discussion is introduced for the results. The characterizations, including EDAX, SEM, and XRD, are carried out to characterize and investigate the chemical composition, microstructure, and crystallography characteristics for CPNC concrete and CPNC mortar. Detailed discussions are presented and shown in Tables 2, 3 and 5 and Figs. 2, 3, 4, 5 and 6 for EDAX, SEM, and XRD, respectively.

#### Mechanical properties testing

The main mechanical properties have been investigated using a standard compression test since the main important mechanical property of the concrete is the compressive strength. The mechanical property testing includes compressive strength, load‒displacement relation, ductility, failure modes, and deformation shape. The fracture mechanics test results for fracture properties include fracture toughness, and fracture energy. The tested samples and test setup are shown in Fig. 2a–c. The results are shown in Tables 1, 2, 3 and 4 and Fig. 2a–c for each mortar and concrete composite. Mortar represents composites of sand only and CPNC, which represents the mortar, while concrete may consist of only coarse aggregate and CPNC or coarse aggregate plus sand and CPNC. Compressive strength, Young's modulus of elasticity € and failure in addition to fracture mechanics properties are developed. As shown in Tables 1, 2, 3 and 4 and Figs. 2a–c and 3a,b, the results of mechanical and fracture properties are indicated fundamentally. The results of the mechanical property tests are shown in Table 1 and Fig. 2a, where Fig. 3a represents the relation of CPNC (HDPE + MMT nanoclay) with pure HDPE, while Fig. 3b represents the load‒displacement of the tested cylinders of CPNC concrete under the unconfined compression test showing the elastic‒plastic behaviour of CPNC concrete with CPNC ratios of 5%, 10%, and 15%, showing the best result for 15% CPNC. Table 1 summarizes the results of the CPNC mortar showing compressive stresses of 2.5, 12.5, and 13.35 MPa), maximum loads of 9, 44, and 47 kN, and compaction ratios of 0.0, 10, and 15 mm, respectively, indicating that 10% and 15% CPNC obtained the best results. At the same time, the modulus of elasticity is 285, 150, 106.5 MPa), respectively, which means that 5% CPNC is most ductile, whereas 10% and 15% CPNC concrete is more elastic‒plastic (brittle‒ductile), which is the best result because pure brittle or pure ductile materials are not required and are not suitable for concrete to avoid large deformation of pure ductile material and avoid sudden cracks and failure for pure brittle materials. The results of the tested samples of CPNC-concrete and CPNC-mortar are summarized in Table 3 and Fig. 3a,b showing the compressive strength of different CPNC ratios (5%, 10%, and 15%). The results show that samples with a 15% CPNC ratio are the best, where the compressive strength is 23.4 MPa), where Fig. 3a shows the elastic‒plastic behaviour of CPNC concrete, while it is known that Portland-cement concrete is brittle^12–17^. The results of the tested beams for fracture properties are indicated in Table 4 and Fig. 4a,b, showing that the best results are of the 15% CPNC ratio. Beams were tested under a three-point load for fracture property testing to investigate the critical fracture toughness (K_Ic_) and fracture energy (G_f_) for both beams made of CPNC mortar (sand + CPNC) and CPNC concrete (coarse aggregate + sand and CPNC with different ratios of CPNC). The test results are in Table 4, showing that the best results are for the beam with a 15% CPNC ratio. This is due to the presence of an additional bond between CPNC and sand producing better interfacial bonding. The SEM micrographs in Fig. 5 for the microstructure of CPNC mortar and CPNC concrete show fine homogeneity with interfacial bonds. Additionally, the results of EDAX analyses in Table 5 and XRD analysis in Fig. 6 prove good homogeneity and particle arrangements without defects or cracks. The results of the mechanical properties, fracture properties, SEM characterization, EDAX analyses and XRD characterization match each other, confirming that the newly developed material can safely replace conventional Portland-cement concrete.

The mechanical properties are investigated through standard tests of cubes and cylinders; standard pre-cracked beams are tested to investigate fracture properties. Newly developed CPNC concrete involves a special processing technique for casting with new factors of compaction, bonding, compressive strength, tensile strength, specific-weight, Young's modulus, ductility and fracture properties; these parameters depend on the properties of the components, ratio, particle size, thermal properties and melting point. An increase in CPNC ratio controls compaction ratio, compressive and tensile strengths, ductility, concrete weight, modulus of elasticity, fracture toughness and fracture energy. An increase in the CPNC ratio increases compaction ratio and vice versa; compressive strength increases due to an increase in CPNC ratio from high bonding between aggregate particles and CPNC particles, which are reinforced polymers with nanoclay particles covering aggregate and sand particles. A change in heights of cubes or cylinders after thermal processing indicates a self-compaction ratio and changes in density and ductility. An increase in CPNC increases the ductility. Self-compaction is developed due to the melting of the CPNC and self-sedimentation of aggregate particles in the melted CPNC matrix based on the ratio of the melted CPNC viscosity concentration to the aggregate particle weight under gravity. Therefore, size and weight values of aggregate particles are very important factors for ductility, strength, E, and mechanical properties of no-cement CPNC-concrete. While conventional concrete requires curing with water for 28 days after casting, the recently developed CPNC-concrete does require any curing or water. For sample preparation, moulds of cubes and cylinders are prepared with a special method based on the thermal casting mechanism and the concrete mix behaviour; the moulds are compacted during heating. The moulds have reduced the heights, but they need their pre-excess heights to match the standard dimensions of cubes and cylinders after compaction. Since it is impossible to increase the height during heating, it is important to increase the heights of the cubes, cylinders and other types of moulds before casting with the expected ratio of height decrease to obtain the required standard height after thermal casting. Height of the sample decreases due to compaction and increases in other mould dimensions; these changes occur due to thermal expansion of the moulds. After cooling the moulds, increase in the mould dimension due to expansion is decreased once again, producing lateral pressure on the samples; this process represents self-prestressing, increasing the compressive strengths and the mechanical and fracture properties of the concrete samples.

#### Fracture properties testing

The fracture properties include fracture toughness (K_Ic_) and fracture energy (G_f_). This section is important for this study. The designer of concrete structures is always aware of cracks for safe design without fracture, cracking or failure and for a sustainable and safe lifetime; Portland-cement concrete is very brittle with easy possible fracture, rapid crack propagation and sudden failure. Therefore, it is necessary to study the fracture properties that control the design to prevent cracks by investigating the main intrinsic parameters of fracture toughness K_Ic_ at crack tips, and the critical fracture energy G_f_. Based on the ASTM standard test for fracture toughness, experimental work is carried out for CPNC-concrete and CPNC-mortar samples.

## Characterization

Characterizations of the chemical composition, microstructure and crystallography are performed using EDAX, SEM and XRD, respectively, to investigate the microstructure, interfacial bonding between particles, particle distribution, particle size and defects for the developed materials: the non-MMT nanoclay, HDPE polymer, CPNC made of MMT and HDPE, CPNC-concrete, and CPNC-mortar.

### Chemical composition characterization with EDAX

The chemical composition of the newly developed thermally cast, no-cement concrete and its components are characterized by EDAX scanning electron microscopy. Table 5 shows the detailed elements of the chemical composition of each of the NC and CPNC samples, including the no-cement concrete nanocomposite materials of CPNC-mortar and CPNC-concrete; the tables indicate the clear differences of the elements between each specimen. The results prove the interfacial bond, microstructure morphology, and crystal and particle sizes before and after producing the composition. The chemical composition is checked by using the EDAX of the scanning electron microscope to check the elements of the composition of the nanoclay, the CPNC made of HDPE and 5% nanoclay platelets, the no-cement concrete nanocomposite made of sand/CPNC and the no-cement concrete nanocomposite with sand and coarse aggregate. The results show the elements and their ratios in the composite.

### SEM characterization

SEM shows the microstructure and particle size characteristics of aggregates and the polymer matrix, shape and distribution characteristics of nanoclay particles; the crystal sizes of nanoclay particles are shown and characterized by XRD based on Sherrer's law (D) and the spacing (d) between atom layers according to Bragg’s law^35^. The SEM micrographs show the microstructure, homogeneity, and distribution characteristics of the particles of the aggregates in the CPNC matrix, the exfoliated, dispersed and intercalated nanoclay particles in the polymer matrix surrounding the aggregate particles, the bonds between the CPNC and aggregates, and the bonds between the nanoclay particles and polymer. SEM micrographs prove the approach of both CPNC and the recently developed thermally cast no-cement CPNC concrete, as indicated in Figs. 1, 2, 3 and 4, and from the results of testing the mechanical and fracture properties, as shown in Tables 1, 2, 3 and 4. Additionally, the results of EDAX prove the concept and mechanism of the newly developed concrete, as shown in Table 5. Additionally, XRD analysis for characterizing the crystal geometries and sizes, as shown in Fig. 6a–d for nanoclay, CPNC, CPNC-mortar and CPNC-concrete, respectively, prove the concept, model, and properties. The microstructure morphology, particle distribution, homogeneity, interfacial bonding, microstructure, dimensions, particle geometry, particle size, CPNC bonding and reinforcing matrix characteristics are shown for the nanoclay, CPNC, and no-cement concrete composites, as characterized by SEM analysis.

### XRD characterization

The crystallography of the newly developed thermally cast, no-cement, no-water CPNC-mortar and CPNC-concrete materials and their components have been characterized by the technique of X-ray diffraction (XRD) based on Bragg's law^35^ and Sherrer's law^36^ to determine the changes in distances between the crystal atomic surface (d) and the particle size before and after processing and the changes in the interfacial bonds between different components and homogeneity; the findings prove the achievement of other properties, such as the mechanical and fracture properties.

### Comparison

Regarding comparing between CPNC-concrete and conventional concrete; CPNC-concrete is a suitable construction material for all types of buildings and infrastructures, including roads and highways, especially in arid and semiarid areas; it does need water for synthesis or curing, and it can be used as a thermal insulator because it contains clay-based polymer nanocomposites, which are thermally resistant and fire retarding functional nanomaterials^12–30^. CPNC concrete is evaluated based on the ACI code and ASTM C 140 to check the water absorption ratio^12–17^. The absorbing water ratio is zero (0.0%), proving that CPNC-concrete is a hydrophobic material due to use of CPNC; Portland-cement concrete^12–17^ is a hydrophilic material that absorbs water. Therefore, CPNC-concrete cannot cause steel corrosion. It has important properties relative to conventional Portland-cement concrete, as shown in Table 6.

## Conclusion

Newly developed eco-friendly green CPNC-concrete and CPNC-mortar are thermally cast, zero-Portland-cement, zero-water mixing, no-crushing, no-brittle fracture, no-water-curing, self-compact, lightweight, low-density, ductile, rapidly processed, rapidly strengthened, and homogenous materials without pores or cavities. These are made of aggregates and nanoclay–polymer nanocomposite (CPNC). CPNC is made of reinforced polymers with nanoclay platelets, such as MMT and halloysite nanotubes. CPNC-concrete is ductile material resists cracking and does not suffer from crushing, sudden brittle failure, or shrinkage cracks. CPNC-concrete has good mechanical and fracture properties. It has good interfacial bonding between CPNC and aggregates, which enhances mechanical and fracture properties. The moulds of developed thermal-casting CPNC-concrete are special metallic moulds with internal removable thermal insulation lining of a very thin layer of aluminium sheets. It has no aggregate-segregation or bleeding as in cases of Portland-cement concrete. It is thermally resistant since it is made of thermally resistant CPNC that are suitable for arid areas. It does not need water; water is rare in arid areas. It is hydrophobic material since it does not absorb wate. It can reduce energy consumption for buildings since it contains CPNC, which is a fire retarding and thermal resistant material that can act as a building thermal insulator for controlling energy transmission. It is rapidly processing with quickly developing strength that does not need as long a time for synthesizing as Portland-cement concrete; Portland-cement concrete needs 28 days to reach 80% of its strength. Moreover, it is economical since it eliminates Portland-cement and water consumption and can use recycled polymer and natural clay**.** In reinforced CPNC-concrete elements CPNC can cover the reinforcement steel, producing high interfacial bonding with concrete and protecting steel reinforcement from corrosion. CPNC-concrete is convenient for 3-D printed structures, especially in areas with few water resources. Finally, CPNC-concrete can resist the spread of diseases due to Portland-cement industry and applications. New special methods of design and construction, special codes are needed for these new materials. It will become a feasible material for future construction projects.

## Data Availability

The data used and/or analysed during the current study available from the (RSH) on reasonable request.
